# Do nutrition-sensitive agriculture interventions work among ethnic minorities in Northern Vietnam amidst the COVID-19 crisis?

**DOI:** 10.1007/s12571-025-01580-2

**Published:** 2025-09-04

**Authors:** Lan Thuy T. Nguyen, Marrit van den Berg, TjeerdJan Stomph, Deborah Nabuuma

**Affiliations:** 1 Development Economics Group, Wageningen University and Research, Hollandseweg 1, Wageningen, 6706 KN the Netherlands; 2 Bioversity International, Penang, Malaysia; 3 Centre for Crop Systems Analysis, Wageningen University and Research, Wageningen, the Netherlands

**Keywords:** Diet diversity, Impact pathways, Agrobiodiversity, Impact evaluation, Social equity

## Abstract

Undernutrition remains a significant challenge among ethnic minorities in Northern Vietnam, possibly due to limited diet diversity. Our study explored the potential of a nutrition-sensitive agriculture intervention (NSA) to improve diet quality among the Thai, H’Mong, and Dao communities using a mixed-methods approach. Conducted between December 2020 and July 2022 during the COVID-19 pandemic, the study involved 600 households across 36 clusters, divided into two treatment groups: one received nutrition and agricultural training, another received this training plus a variety of vegetable seeds, and a control group without any intervention. We focused on diet and crop diversity, especially in vegetables and legumes. Quantitative data were collected through one baseline and two end-line rounds to ensure sufficient statistical power, while qualitative data included 14 focused group discussions, seven in-depth interviews, and field notes from field assistants. Our findings revealed that diet diversity, particularly in vegetables and legumes, was limited among the H’Mong and Dao, underscoring the potential of NSAs to improve their diet. Market access and on-farm production were found to complement each other in enhancing both diet and crop diversity across all three communities. The impact of the NSA intervention highlighted the complexity of contextual factors, including initial conditions and the ongoing effects of the COVID-19 pandemic, which influenced the outcomes in unpredictable ways. Nonetheless, combining seed provision with nutrition and agricultural training emerged as a promising strategy to enhance both diet and crop diversity, particularly given the strong vegetable cultivation practices and limited market access in these communities.

**Supplementary Information:**

The online version contains supplementary material available at 10.1007/s12571-025-01580-2.

## Introduction

Despite substantial global advancements in food security and nutrition security in recent decades, the persistence of undernutrition poses a significant health and developmental challenge, casting doubt on the realization of the `zero hunger’ goal outlined in the Sustainable Development Agenda for 2030 (​FAO et al., 2022). Particularly, ethnic minority populations experience disproportionately higher rates of undernutrition compared to their ethnic majority counterparts in the same regions or countries (Horta et al., 2013; Mas-Harithulfadhli-Agus et al., 2021; Wang & Mashford-Pringle, 2022). This disparity is rooted in the profound political and socioeconomic marginalization faced by these groups across all countries where they reside (Broaddus-Shea et al., 2020; Kabeer, 2006).

While Vietnam has made notable progress in alleviating poverty and enhancing food and nutrition security over recent decades, the ethnic minorities continue to grapple with persistent undernutrition (Harris et al., 2021). In this country, the highest stunting rates and micronutrient deficiencies were observed among ethnic minorities (Mbuya, 2019). According to the National Nutrition Survey 2020, the stunting rate among children aged 2 to 4 years in mountainous areas—where ethnic minorities are primarily located—was 34.1%, more than double the national average of 15%. Additionally, the prevalence of iron deficiency among children aged 5 to 9 years in this region was twice as high as the national average (Tan et al., 2024). One of the underlying reasons for this is their starch-based diet with a limited number of rich sources of micro-nutrients (Eckhardt, 2006; Nguyen et al., 2013).

Meanwhile, diet diversity is crucial to battling undernutrition since maintaining a healthy body requires the consumption of a wide variety of nutritious meals, combining different vegetables, fruits, legumes, and animal derived foods (Jones, 2017). Expanding the composition of diets to incorporate a wider diversity of food items promotes the intake of essential nutrients, such as micronutrients and dietary fibers. By including a broader selection of foods, the overall nutritional quality of the diet is enhanced, which leads to improved health outcomes and better support for bodily functions (Korir et al., 2023; Zimmerer & de Haan, 2017).

Nutrition-sensitive agriculture interventions (NSAs) have been reported to demonstrate strong potential to improve the diet diversity of targeted groups (Ahmed et al., 2023; Sharma et al., 2021). NSAs include projects such as nutrition education, together with other programs promoting agrobiodiversity systems, or promoting biofortified crops. NSAs have potential, particularly in rural areas where agriculture is central to providing food, livelihood, and income for households. Empirical studies have revealed positive correlations between diet diversity, agricultural production diversity, nutrition knowledge, and women’s empowerment (Ruel et al., 2018; Sharma et al., 2021).

In this study, we used quantitative and qualitative data from a Randomized Control Trial (RCT) of an NSA implemented among ethnic minorities in Northern Vietnam to understand how, and under what conditions, NSAs can improve diet diversity. Specifically, this paper aims to address the following two main objectives. First, we investigated the crop diversity and diet diversity in our study context. Second, we scrutinized the impact of an NSA on diet diversity and crop diversity of ethnic minority groups in Northern Vietnam during the COVID-19 pandemic. The NSA included two treatments (1) Nutrition and agriculture training, and (2) Seed provision.

We contribute to the literature in three ways. First, we improve the understanding of diet diversity and crop diversity among ethnic minorities, which are facing chronic undernutrition issues (Mbuya, 2019). However, they are also recognized for their rich access to agrobiodiversity, that typically contributes to a diverse diet (Hanh, 2006; Jones, 2017). Their landscape is known for conducive conditions for multiple crops rich in Vitamin A and Iron, which are beneficial for fighting vitamin A deficiency and anemia, both with high prevalence in the studied regions (Mbuya, 2019). Our focus was on addressing diet imbalances in vegetable and legume food groups, specifically targeting dark green leafy vegetables (DGVL), other Vitamin-A rich vegetables^1^, and legumes^2^, as the food groups most relevant for women’s health out of the 10 food groups from the FAO classification (FAO, 2021). Furthermore, these foods contain a high amount of other vital vitamins, minerals, and antioxidants that are important for health. Legumes, in particular, are considered as affordable and sustainable protein sources (Iqbal et al., 2006; Semba et al., 2021; van der Walt et al., 2009). Given this context, it is crucial to investigate further the agricultural practices and dietary habits of the targetted households to understand the current state of their food diversity.

Second, we enhance our understanding of how NSAs can (or cannot) achieve the intended outcomes. In a comprehensive summary of 85 studies, Di Prima et al. (2022) showed that NSAs are subject to various highly context-specific factors that may interact with the intervention design and implementation. These interactions increase the unpredictability of the outcomes and expected mechanism of impacts,affecting efforts to effectively scale these interventions. Therefore, the exploration of these programs in new contexts becomes not only relevant but also integral to advancing our knowledge of NSAs. Our study context is unique in the literature for two reasons. First, there is an underrepresentation of ethnic minorities in NSA literature (Sharma et al., 2021). Second, the COVID-19 pandemic marked an unprecedented time in our history. During this period, social distancing and market closures led to reduced income from product sales, combined with increased food prices due to supply shortages (Agyei et al., 2021). COVID-19 measures also impeded access to inputs and agricultural services, resulting in reduced food production and limited on-farm food availability (Harris et al., 2020).

Third, we address the knowledge gap regarding how seed provision interventions can (or cannot) enhance the effectiveness of nutrition and agricultural training. Providing bundled interventions has been an advised practice for NSA designers, and providing agriculture inputs, including seeds is the popular choice (Mayorga-Martínez et al., 2023; Nabuuma et al., 2022). Nevertheless, the mechanisms by which seed provision reaches nutritional targets remain underexplored. This knowledge is crucial for practitioners to determine which types of interventions should be included to effectively improve production and consumption diversity, and under what conditions.

## Study settings

Vietnam is a multi-ethnic country with 54 different ethnic groups. The largest of these is the Kinh (or Viet) ethnic group, comprising 85% of the total population (GSO, 2019). The Vietnamese government uses the term “ethnic minority” in reference to all groups except the Kinh majority. Except for the Hoa (ethnic Chinese), which are well assimilated into the Kinh’s culture and integrated into the Kinh community, ethnic minorities distinguish themselves from the Kinh and each other through unique languages and various distinctive cultural practices. These minority communities represent the most economically challenged segment of the population, with 45% living below the national poverty line in 2016, compared to a mere 3.1% of the majority group (Pimhidzai, 2018). Many groups also live in remote areas like the mountains in northern Vietnam. To reduce the poverty rate in these mountainous areas, the government has introduced numerous infrastructure development programs to boost the regional economies (Socialist Republic of Vietnam, 2010). However, these projects were criticized as disproportionately benefiting the majority population rather than ethnic minorities (Nguyen & Rama, 2007). Besides, the minorities constantly face social discrimination and prejudice from the majority, which holds national authority (Choi, 2014; Nguyen, 2021). As a result, they encounter exclusion from market access, education, and healthcare services (Harris et al., 2021; Turner, 2012; Van de Walle & Gunewardena, 2001).

**Fig. 1:**
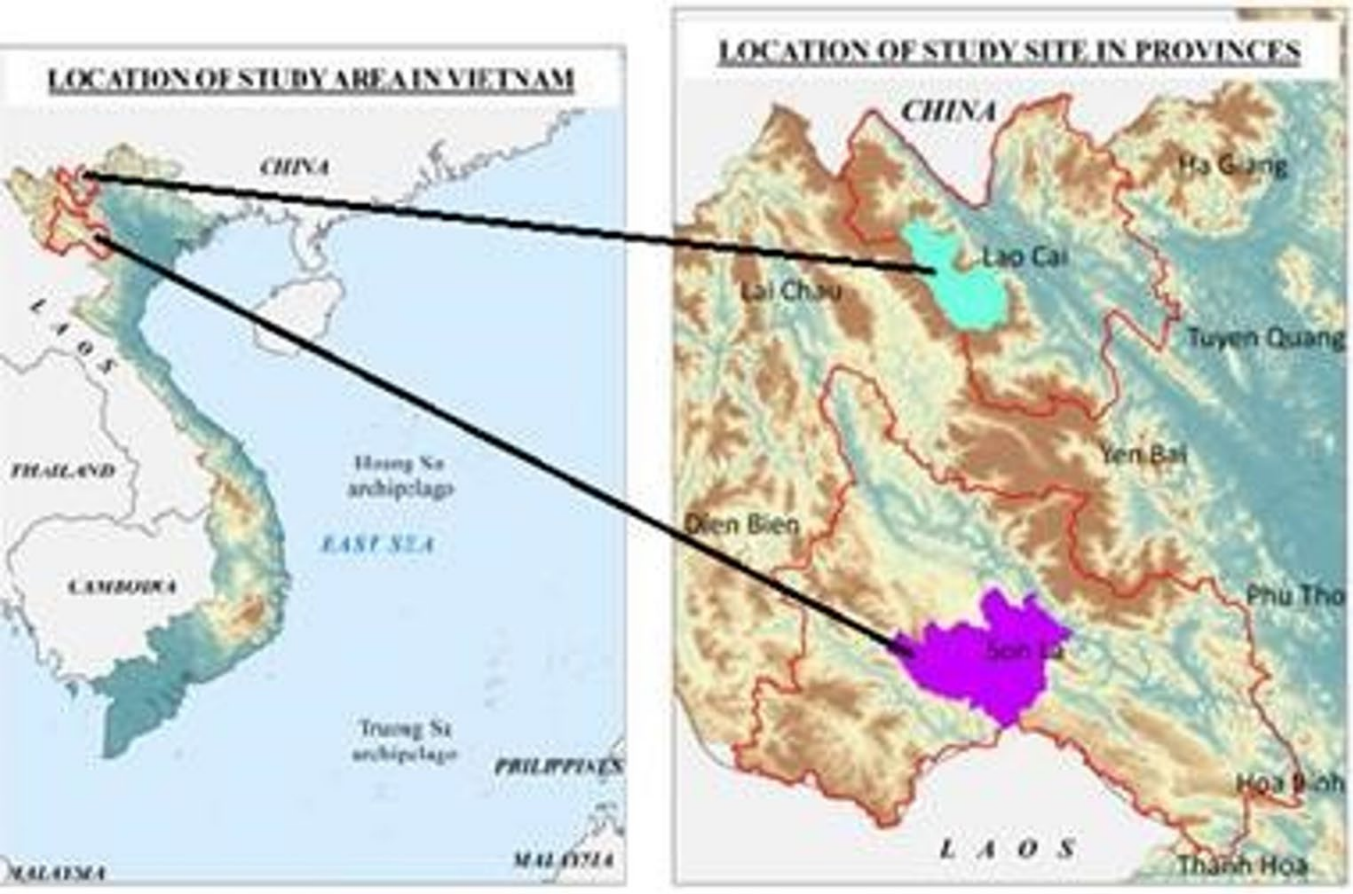
Study regions

The current study was conducted from 2020 to 2022 in 38 villages, organized into 36 experimental clusters, in Mai Son district, Son La province, and Sa Pa district, Lao Cai province in northern Vietnam (Figure 1). These regions are well-known for being home to a substantial population of various ethnic minorities, including H’Mong, Thai, and Dao (ADB, 2015).

The infrastructure, particularly the road networks connecting the village centers and district townships are generally well-developed in both regions. The road conditions in Thai villages tend to be more developed than in some H’Mong and Dao villages, and several Dao and H’Mong households, especially in Sa Pa, experience significant isolation. This is largely due to the steep and rugged mountains in Sa Pa, which make road construction challenging. However, many villages have grocery stores or mobile street vendors offering specific fresh food items, drinks, and sweets, on top of active commune and district markets.

The climatic conditions of both study sites are highly favorable for horticultural activities. Particularly, the climate in Sa Pa is conducive to horticulture and is suitable for growing temperate vegetables, providing a unique condition in this otherwise tropical country. Traditionally, ethnic minority households cultivate various kinds of vegetables and legumes for home consumption in their home gardens or by intercropping with annual staple crops. These conditions suggest high potential benefits of implementing NSAs to promote diet diversity among these groups.

## Materials and methods

### Description of the NSA

The basis of the NSA involved the establishment of Community Diet Diversity clubs aimed at encouraging the consumption of diverse diets with an increase in crop-based food of three targeted food groups: dark green leafy vegetables, other vitamin A-rich vegetables, and legumes. These clubs were established in 18 randomly selected clusters out of a total of 36. Within each selected cluster, we randomly invited twenty to thirty households to nominate a member knowledgeable about vegetable and legume production and food preparation to join these clubs. Notably, a majority of club members were women and illiterate, likely influenced by the traditional association of nutrition, vegetable production, and food preparation with women in the region.

Club members participated in one or two treatments. The first treatment, open to all Club members, was an agriculture and nutrition training program consisting of eight sessions. These sessions covered a range of topics, including diet diversity, food groups, essential micronutrients like Iron and Vitamin A, participatory cooking, vegetable and seed production, food shopping, and managing a food budget.

Given that literacy could be a significant barrier to knowledge acquisition among club members, we implemented targeted strategies to enhance message retention and comprehension. First, training materials were designed to be accessible to non-literate individuals. These materials includes audio podcasts in the members’ native language, visual agriculture and nutrition posters summarizing key respective messages, and booklets detailing the benefits of the key food groups and simple, healthy recipes. The booklet were developed in response to participants’ requests. This included primarily image-bases with some Vietnamese text, with culturally sensitive layout with ethnically imagery to enhance engagement and relatability (Ngoh & Shepherd, 1997). Second, each participant received summary posters and a booklet to take home. They were encouraged to display these materials in their living spaces and discuss them with literate peers or family members to reinforce learning through knowledge-sharing.

The training curriculum mainly focused on nutrition and diets, covered in three theoretical sessions lasting 45–90 min each and two participatory cooking sessions lasting for 90–120 min each. Additionally, there were two sessions on vegetable and seed production, each lasting 30 min with an additional 30 min dedicated to practical exercises. One session focused on food shopping and managing food expenditure, lasting 45 min. The training sessions were designed to be interactive, featuring activities like listening to audio podcasts in members’ native language, discussing key messages, playing games, and participatory cooking facilitated by field assistants from the Alliance of Bioversity International and CIAT and local facilitators. After listening to the podcast, facilitators reinforced key messages using posters, with interactive discussions throughout. In participatory cooking sessions, participants were required to bring ingredients belonging to the key food groups. The training frequency was planned as once per month.

The curriculum and training materials were developed by three research team members, one is also co-author of this paper, two of the three have extensive experience in managing other nutrition-sensitive agriculture intervention projects. The materials were further updated and developed according to the suggestions of club members.

**Fig. 2:**
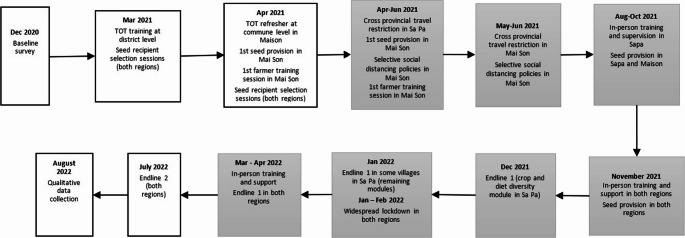
Study timeline. Grey blocks represent the major outbreak period of the Covid - 19 pandemic in the study regions

Treatment two was an add-on to the first treatment, focusing on improving club members’ access to quality and diverse seeds. This treatment involved the distribution of various vegetable and legume seeds to a randomly selected half of the club members, while the remaining half received cooking spices and soap of equivalent monetary value.

Using baseline survey data, in each region, the research team identified nine vegetable and legume crop types^3^ representing the three key food groups. These crop types were the least consumed key crop types according to our baseline data, and should not require unfamiliar cultivation techniques. We then consulted our selections with the communal leaders. While this approach may not fully reflect farmers’ preferences as a participatory method would, it streamlined the process by reducing the time and effort needed to consolidate diverse demands across villages given our resource constraints. Our current approach may limit the potential impact of seed provision compared to a participatory approach, which indicates that the estimated treatment effects presented our study are conservative and should be higher in future projects.

However, farmers still had some choices, as they were allowed to select seeds of five crop types from the given list of nine. This strategy not only enabled some degree of preference expression but also increased the perceived value of the seeds by making opportunity costs more salient. In turn, this helped mitigate potential spillover effects from seed sharing between recipients and non-recipients (Makarina et al., 2019). Self-reported data at endline confirmed that seed sharing was infrequent, with only 2% of seeds reported as shared.

Seed recipients received individual supervision and assistance through phone calls or home visits from field assistants. Seed distribution was planned for the two weeks prior to the planting seasons. Details on the provided seeds and provision timeline can be found in Table 1, Appendix 1.

The trainers leading the training sessions were primarily selected from the leaders of the village’s women’s union, with village chiefs also taking on this role in some cases. The selection process for these trainers was conducted by the commune Women’s Union. To prepare the trainers for their role, the research team trained them through two Training of Trainer (TOT) workshops on the training modules and group management skills. Both workshops covered the same topics to ensure that the trainers were sufficiently trained. For each training session, trainers received reimbursement equivalent to the average local salary for one working day, and the club members were reimbursed for their travel costs only. Throughout the project implementation, the trainers received support from two field assistants recruited by the Alliance of Bioversity International and CIAT. These assistants, recent bachelor’s degree graduates, were familiar with the study site context and did not face major language barriers with the trainers.

### Expected mechanisms of impact

Through these treatments, we anticipated that the club members would increase their consumption of the key food groups, driven by two main mechanisms. First, enhanced knowledge about the health benefits of diet diversity, including the specific nutrient properties and health advantages of the key food groups, was expected to stimulate greater demand for these foods. This heightened interest would likely lead to changes in consumption patterns, either through increased purchases of the key food groups or through consumption of own produce. Second, the seed provision was designed to improve access to seeds of the key food groups, thereby leading to greater crop diversity. As a result, seed recipients were expected to enhance their consumption of these key food groups, thanks to the increased availability from their production. In short, this dual approach aimed not only at improving diet diversity through educational outreach but also by ameliorating crop diversity.

### COVID-19 policy responses in Vietnam

Our intervention lasted for 10 months from March 2021 to January 2022, amid the challenges posed by the COVID-19 pandemic in Vietnam (Fig. 2). During this period, the national government adopted a distinct track and trace strategy in response to the pandemic. The core objective of this policy was to isolate individuals who either tested positive for COVID-19 or were deemed high risk due to close contact with infected individuals or travel from highly affected areas. These identified individuals were mandated to either self-quarantine at their homes or stay in designated quarantine facilities for a duration of 7 to 14 days (Nguyen et al., 2021). The rationale behind this approach was to circumvent the need for broad social distancing protocols, focusing instead on high-risk groups and communities. This strategy aimed to allow the larger economy to continue operating with minimal disruptions.

However, the implementation of this policy inadvertently resulted in significant social stigma towards those identified as high-risk. This stigma manifested in widespread reluctance among many individuals to participate in social gatherings or travel, driven by a fear of being subjected to judgment, blame, and discrimination from their communities (Trinh et al., 2022). Over time, as the number of cases rose and more information about the virus’s effects became available – revealing less severe health impacts than initially feared – the stigma associated with the virus gradually subsided.

The COVID-19 pandemic also impacted our study regions, with Mai Son experiencing more severe outbreaks than Sa Pa (Appendix 3). Between April and December 2021, the primary project implementation period, at least one study village in Mai Son reported an infection each month, whereas Sa Pa recorded few close contact cases only during the same period. Despite the lower infection rates in Sa Pa, the project team encountered equivalent challenges in both regions. Local authorities in Sa Pa discouraged inter-commune travel, leading to delays in seed and material procurement. Meanwhile, in Mai Son, travel restrictions prevented field assistants from providing in-person support, further complicating intervention implementation.

Amid these complexities, the project team maintained a strong connection with the local context, demonstrating flexibility by adjusting the training schedules in coordination with local authorities and facilitators. We managed to conduct all training sessions in the curriculum in person while adhering to safety protocols from the government. We also offered online assistance to complement the in-person support for local trainers and club members, especially during times when villages experienced isolation due to the aggressive spread of the virus (Fig. 2). However, several of the planned practical trainings on vegetable and seed production had to be dropped due to the trainers’ preference for shortening the trainings, and logistical difficulties in organizing practical sessions with online assistance. The flexible training schedule indicated that the gap between some sessions were longer than intended. Furthermore, the delay in seed provision in Sa Pa made the optimal planting seasons of some crop types were missed, which exert implications on the interventions’ effects.

### Data collection

#### Quantitative components

Our quantitative data consists of one baseline survey conducted in December 2020 and two endline surveys, administered between December 2021 and March 2022, and in July 2022, respectively (Fig. 2). To enhance statistical power, we implemented an additional endline survey round. This approach is cost-effective when the expense of expanding treatment to new clusters exceeds the cost of conducting additional survey rounds (McKenzie, 2012). With this design, our minimum detectable effect sizes (MDE) at 80% power for crop and diet diversity indicators range between 4% and 20% 4.

We believe that the seasonal differences between baseline and endline measurements do not pose significant biases on our treatment assignment. As treatment assignment was conducted prior to baseline survey, thus independent of post-treatment outcomes, and stratified by ethnicity and distance from the commune to the closest district market, both treatment and control groups were subject to the same seasonal patterns. To further account for potential seasonal variations, we included baseline outcomes in our treatment effect estimation model. This adjustment helps control for unobserved variables highly correlated with seasonal changes in crop diversity, such as soil conditions and individual crop preferences, as suggested by Lüdtke and Robitzsch (2023).

The baseline survey included 584 households from 36 villages. We randomly selected 10–15 households in control clusters, and 20–25 in the treatment clusters. The details of sampling calculation and randomization protocol can be found in Appendix 2. Respondents were chosen among household members responsible for and knowledgeable about crop production and food preparation. Most respondents were women. During the baseline interviews in treated villages, participants were invited to become club members or to nominate another household member to participate in the training. This invitation was reiterated by local facilitators before the first session.

The survey questionnaire comprised four main sections: nutrition knowledge; diet diversity; vegetable and legume production; and demographic information. We assessed nutrition knowledge through a series of questions on diet diversity and micronutrients, with a maximum achievable score of 20. Diet diversity was measured by the number of key food groups consumed and the number of key types consumed from market purchase and on-farm production combined. These indicators were captured individually and evaluated through two methods: a 24-hour quantitative diet recall for all food groups and a 7-day qualitative diet recall focusing on vegetable and legume food groups only. In the 24-hour diet recall, we also measured the food quantities in grams, using the ‘Food Atlas’ developed by the Vietnam National Institute of Nutrition (NIN) which provides visual references for portion sizes^5^.

Crop diversity was assessed based on the number of key food groups and key crop types cultivated. We employed a simple count method, which aligns with diet diversity frameworks where overall food availability is prioritized over crop distribution within farms (Keding et al., 2012) These indicators were collected by asking respondents to list all vegetables and other crops grown in the past three months and the seed source of each of these. FAO’s guidelines for food group classifications to compute minimum diet diversity for women were followed for the computation of the crop and diet diversity indicators (FAO, 2021). To the best of our knowledge, this study is the first to apply FAO’s classification to measure crop diversity at the food group level in this context. Previous studies in the same setting have primarily assessed crop diversity at the species or cultivar level (e.g. Genova et al., 2022; Ha et al., 2019).

After the baseline survey, adjustments to administrative boundaries affected several villages of the H’Mong people in Sa Pa, resulting in 23 households being situated outside of the designated village areas. Furthermore, the proximity of two sets of two villages raised concerns about potential spillover effects. In response, we randomly enlisted new households to substitute those administratively excluded, made clusters of the nearby villages, and incorporated two additional villages so that the number of clusters remained 36. Consequently, 58 households were newly recruited. Both new and administratively excluded households were incorporated into our sampling frame for the endline. During the endline survey, interviews were conducted with all household members who had either participated in the baseline survey or attended at least one training session, resulting in numerous households having multiple members interviewed.

For the endline surveys, we utilized the same questionnaires as used in the baseline, with specific adjustments made to enhance their relevance and effectiveness. Demographic information was collected exclusively during the first endline survey. This decision was based on the expectation that these variables would have limited change over the three or four months between the two end-line rounds. Additionally, a shorter questionnaire was expected to reduce the attrition rate.

In the first endline round, we collected new data on total farm size and market access to replace the information collected in the baseline. During the baseline survey, the enumerators reported that multiple respondents provided arbitrary estimates of total farm size and that respondents confessed that farm size and distance to markets were beyond their knowledge. It was also mentioned that in this context, men generally make more accurate estimations of their farm sizes. Furthermore, following the guidance of local partners, men were asked as, thanks to their greater mobility, they could provide more reliable estimates on the walking time from home to the district market and the closest fresh food market.

The enumerators were from the same ethnicities as the respondents or spoke the languages the respondents were fully proficient in to avoid translation bias and loss of any cultural nuances that could hamper data quality.

The research team trained them for at least two days, including a 1.5-day in-door training and a half-day piloting. Several enumerators joined more than one survey. The data were collected using ODK software in the baseline and SurveyCTO software in the endlines. This was a pragmatic choice based on availability and since both are similar digital data collection tools, was considered not to affect data consistency.

#### Qualitative components

A qualitative survey was conducted in August 2022, together with a qualitative study on women’s empowerment under the same umbrella project. The primary objective of the qualitative component was to comprehend the implementation process of the interventions and shed more light on the plausible mechanisms of impact. The 18 treated clusters were classified into seven blocks based on ethnicity, district, and distance to the markets. Based on our field observations, we expected that the outcomes would vary by these three variables. We chose one cluster per block and organized two focus group discussions (FGDs) for two types of beneficiaries: training members who received no seeds and those who received both training and seeds, and one interview with village trainers. The discussion topics are summarized in Table 1. Additional in-depth beneficiary interviews were conducted if interesting stories emerged from the focus group discussions. Each focus group discussion had four to six participants, one facilitator, one note-taker, and if necessary one translator. In total, we organized 14 focus groups, seven interviews with village trainers, and three follow-up interviews with individual beneficiaries. Our qualitative data also include field reports from field assistants.

The facilitators were experienced research assistants and the majority of them were from the study regions and were not involved in the NSA before. They underwent a three-day training workshop, along with interview guides on women’s empowerment. The interview and discussion instruments were tested and refined after three trial interviews. To avoid any biases, field assistants, who were exposed most to the local trainers and beneficiaries, were not present throughout the data collection.

**Table 1:** Description of discussion themes in focus group discussions and interviews during qualitative data collection

Tools	Discussion themes
**Focus group discussions (FGDs) with training beneficiaries and seed recipients**	- Perception of project implementation - Barriers to participation - Perception of intervention’s effectiveness on diet diversity and vegetable production - Exploring food market purchase practices
**Interviews with local trainers**	- Perception of project implementation, including training organization and farmer mobilization - Perception of intervention’s impact and its sustainability

### Data analysis

#### Quantitative data

We first checked the balance between treated and control groups by running the following regression:

1 $$Y_{ij}^0=\alpha+\rho_1T_{1j}+\rho_2T_{2j}+u_{ij}$$

Where $Y_{ij}^0$ is a vector of baseline characteristics of individual i in cluster j and 𝑇_1j_ is a treatment dummy for receiving training, and 𝑇_2j_ is a dummy for receiving seeds in addition to the training.

Intent to treat (ITT) treatment effect analyzes the subjects according to their original treatment assignment based on the randomization protocol. This method helps to overcome any biases caused by non-compliance when subjects refuse to take the offered intervention. It will inform policymakers about the average treatment effect on the targeted groups, as dropping out is very common in development projects.

We use the ANCOVA method for ITT estimation. As suggested by McKenzie (2012), this method is more effective than the Difference in Difference method in boosting statistical power:

2 $$\:{Y}_{ij}^{1}=\alpha\:+{\beta\:}_{1}{T}_{1j}+{\beta\:}_{2}{T}_{2j}+{\theta\:}_{1}{Y}_{ij}^{0}+{\rho\:}_{1}{X}_{ij}+{\rho\:}_{2}M{A}_{ij}+{\epsilon\:}_{ij}$$

In which $\:{Y}_{ij}^{1}$: is a vector of endline outcomes where observations of the first and second endline are pooled. $\:{\beta\:}_{1}$ is the treatment effect of the training compared to the control group, $\:{\beta\:}_{2}$ is the treatment effect of the seed provision compared to the training only group.

𝑋𝑖𝑗 are covariates, including household size, wealth index, education of the household head, education of the respondent, age of the household head, age of the respondent, and farm size. The wealth index is the first component extracted from a Principle Component Analysis (PCA) on a list of substantial possessions and big livestock such as cows and buffalos. All covariates were collected at baseline, excluding land area and market access which were collected at endline.

𝑀𝐴_𝑖𝑗_ is access to the district and food market, which was measured by the travel time of the respondents by foot to the closest food market. The food market was defined as the place where respondents can buy at least three out of five food groups, including meat, fish, eggs, vegetables, and Legumes. Our research team developed this definition to align with the study’s focus on diet diversity. Unlike markets that primarily offer staple grains or processed foods, those meeting our criteria better support access to essential macronutrients and micronutrients. This ensures our analysis captures environments where individuals have practical access to a diverse diet, rather than just general food availability.

In this study, we fit equation (1) using three different strategies given the differences in sample composition between the baseline and endline. We first used only respondents who participated in the baseline survey (model 1). Second, we employed the endline sample without adjusting for the baseline outcomes (model 2). This model allows us to utilize the observed information of all endline sample, including newly recruited households after the baseline survey. Third, we analyzed the imputed endline data, adjusting for both the imputed baseline outcomes and the presence of missing baseline data (using a missing baseline dummy variable) (model 3). For the second and third models, we controlled for the status of households at endline, whether they were newly recruited or administratively excluded. In all models, standard errors were clustered at the training assignment level. The results of model 1 will be presented in this paper, while the remaining two models will serve as a robustness check, and their results can be found in Appendix 5.

To further verify our results, we estimated regression (2) using the Bayesian framework with flat prior N~(0,10,000). We compared the confidence intervals of the regression (2) and credentials intervals obtained from the Bayesian approach to detect deviations. The Bayesian framework is recommended to increase the precision of treatment estimation in studies with small sample sizes (van de Schoot & Miočević, 2020).

We proposed a broader range of outcome variables in the Registered Pre-Analysis Plan (PAP)^6^, including the total individual diet diversity scores, and the diversity and quantity of consumed items from the vegetable, legume, and fruit food groups. Our initial idea was to test for spillover effects on food groups that were not the focus of our training curriculum. However, given the hurdles encountered during the implementation process (Fig. 2), we expected that our study would be underpowered to detect such spillovers. Therefore, we decided to analyze the outcomes of the three key food groups only, and only investigate the effects on other food groups when treatment effects were detected in the key food groups to understand further the mechanism of impact.

Regarding missing data, we ignored these if the rate was lower than 5% and imputed missing values otherwise. With this principle, only the variable nutrition knowledge required correction as the missing rate was 12.3% for the baseline sample. To handle the missing data of nutrition knowledge, we filled in missing values using multiple imputation procedure in STATA^7^, using household and individual characteristics.

In addition to ITT effects for the total sample, we conducted heterogeneity treatment effect estimations for the Thai and H’Mong groups. The primary goal of this analysis is to examine how different ethnic groups respond to the interventions and to gain deeper insight into the mechanisms driving the observed treatment effects at the total sample level. This analysis does not seek to establish causal relationships at the ethnicity level due to limited statistical power. The Dao group was excluded from this analysis, given their small sample size (N = 76). As this group possessed distinctive characteristics, which will be presented in detail in the `Results and Discussion’ section, it is not optimal to pool this group with either the H’Mong or the Thai group. Due to the Dao group’s relatively small size compared to the other groups, this exclusion should not affect the main objectives of the analysis.

To explore further the mechanism of impact, we also analyzed the Local Average Treatment Effects (LATEs) of the nutrition training. This involved the Instrument variable (IV) method, in which the actual training participation was instrumented by training assignment. This analysis was carried out exclusively using the estimation model baseline sample, correcting for the baseline outcomes (model 1).

We applied the Romano-Wolf procedure to correct for multiple hypothesis testing (Clarke et al., 2020). We used 3000 replications and corrected for three families: diet diversity, including all indicators collected by the 24-hour diet and 7-day diet recall methods, food quantity, and crop diversity, which comprised all indicators on vegetable and legume cultivation.

#### Qualitative data

The qualitative data were manually coded and analyzed using Atlas.ti. The coding framework used for qualitative data analysis was structured into two main themes: (i) *Intervention Implementation* and (ii) *Perceived Effects*. Each theme consisted of parent codes, which represented broader categories, and corresponding child nodes, which capture specific subcategories (Table 2).

Within *Intervention Implementation*, two parent codes were identified: *Training Organization* and *Seed Provision*. The *Training Organization* category included subcodes related to logistical aspects, such as training schedules, curriculum, materials, facilitators, and barriers to participation. The *Seed Provision* category encompassed aspects such as seed distribution schedules, types of seeds provided, seed quality, and technical barriers encountered.

The *Perceived Effects* theme explored both the intended and unintended consequences of the interventions. It was divided into two parent codes: *Training* and *Seed Provision*, each of which was further categorized into *Intended Effects* and *Unintended Effects*.

This coding structure was applied at the total sample and ethnicity levels.

**Table 2:** Coding tree description of the qualitative data

Themes	Parent codes	Child nodes
Intervention implementation	Training organization	Training schedule Training curriculum Training materials Facilitators Barriers to participation
	Seed provision	Seed distribution schedules Types of seed provided Seed quality Technical barriers
Perceived effects	Training	Intended effects Unintended effects
	Seed provision	Intended effects Unintended effects

### Ethics

The study obtained Ethical Approval from Hanoi University of Public Health, IRB No 428/2020/YTCC-HD3, issued on 4th December 2020, and from Wageningen University and Research, issued on the 1 st February 2021. Oral informed consent was obtained from each participant before both data collection and intervention implementation. During data collection, participants were informed about the study’ purpose, confidentiality, rights of participant, reimbursement and contact numbers. For the intervention, consent included details about the study, the purpose of the intervention, participant rights and contact information. A pre-analysis Plan was registered in the EGAP Registry dated December 18, 2021.

## Results and discussion

### Descriptive statistics

#### Household and individual characteristics

The descriptive statistics of household and individual characteristics of the baseline sample are reported in Table 3. In our baseline sample, 86% were women. Households were characterized by small landholdings, with an average total farm size of 1.81 ha (SD = 1.54). Additionally, both respondents and household heads had limited formal education, and respondents were less likely to have completed primary school compared to the household head. On average, respondents were around 40 years old (SD = 11.6) and lived in households with approximately five members (SD = 1.76).

Notably, this household size exceeded the national average of 3.6 members in 2020 (GSO, 2021). Geographically, our baseline sample tended to reside at a considerable distance from the closest district market, necessitating an average walking time of 2.84 h (SD = 1.62) to reach the closest district market. Despite of this distance to district markets, households still had access to fresh food markets, with the closest one reachable within an average walking time of 0.36 h (SD = 0.42) (Table 3).

The characteristics varied significantly across different ethnicities (Table 3). Compared to the H’Mong and Dao, the Thai were significantly better off with larger farm sizes and higher education levels of both household heads and respondents. Meanwhile, the H’Mong had the largest household size with 0.46 (SE = 0.17) more members than the Thai, and they generally lived closer to the district market. Among the three ethnicities, the Dao was the most geographically isolated, facing the greatest distance to both district and fresh food markets.

**Table 3:** Mean (SD) of household and respondent characteristics for the total sample and Thai sample at baseline, and coefficients (SE) from regressions between dependent variables and ethnicities, where the Thai were the reference community and the coefficients for h’mong and Dao indicate differences with the Thai

Dependent variables	Baseline sample (*N* = 584)	Thai (*N* = 217)	H’Mong (*N* = 291)	Dao (*N* = 76)
	Mean (SD)	Mean (SD)	Coefficients (SE)	Coefficients (SE)
The household head is female	0.07 (0.24)	0.08 (0.26)	−0.02 (0.02)	−0.01 (0.03)
Respondent is female	0.85 (0.36)	0.86 (0.35)	−0.03 (0.04)	0.04 (0.06)
Household head completed primary school or higher	0.31 (0.45)	0.44 (0.49)	−0.19*** (0.05)	−0.30*** (0.04)
Respondent completed primary school or higher	0.25 (0.43)	0.39 (0.49)	−0.23*** (0.04)	−0.18*** (0.07)
Age of Household head	45.2 (11.7)	46.7 (11.0)	−3.4*** (1.2)	1.8 (2.2)
Age of respondents	39.5 (11.6)	40.9 (11.2)	−2.6** (1.3)	−1.2 (1.6)
Wealth index	0.01 (1.85)	1.59 (1.66)	−2.48*** (0.26)	−2.69*** (0.25)
Number of household members	5.49 (1.77)	5.26 (1.64)	0.46** (0.17)	−0.01 (0.29)
Walking distance to the nearest district market (hours)	2.84 (1.62)	3.19 (1.14)	−0.91** (0.37)	0.84*** (0.26)
Walking distance to the nearest fresh food market (hours)	0.36 (0.42)	0.22 (0.17)	0.06 (0.04)	0.86*** (0.22)
Total farm size (ha)	1.81 (1.54)	2.39 (1.66)	−0.99*** (0.25)	−0.65*** (0.22)

(1) Coefficients, Robust standard errors, and p-values were retrieved from the model (1) using baseline sample, where 𝑇_1_ is a treatment dummy for receiving training, and 𝑇_2_ is a dummy for receiving seeds on top of the training

(2) *p-value < 0.1; **p-value < 0.05; ***p-value < 0.01.

(3) Standard errors in parentheses are clustered at the training assignment level

#### Outcome variables

Table 4 presents the descriptive statistics of outcome variables at baseline for the total sample and disaggregated by ethnicity. Our study reveals a generally low overall diet diversity score (DDS), particularly diet diversity of vegetable and legume food groups. At baseline, our respondents consumed 3.5 (SD = 1.17) out of 10 food groups based on the FAO classification in the past 24 h (Table 4). Among the three ethnic groups, the Thai had the highest DDS (Mean = 4.2, SD = 1.02), which was closest to the national average of 4.4 food groups (Nguyen et al., 2023). Nevertheless, they did not achieve the recommended minimum diet diversity for women of reproductive age (15–49), which is 5 out of 10 food groups (FAO, 2021).

**Table 4:** Mean and (SD) of baseline total diet diversity, and vegetable and legume types grown and consumed and each food source or seed source of the total sample and for the Thai group; and coefficients (Robust SE) of the regression from each dependent variable and ethnicity dummy for the h’mong and Dao groups, where the Thai group was the reference

Dependent variables	Total sample (*N* = 584)	Thai (*N* = 217)	H’Mong (*N* = 291)	Dao (*N* = 76)
	Mean (SD)	Mean (SD)	Coefficients (SE)	Coefficients (SE)
Nutrition knowledge score	1.44 (2.9)	2.97 (3.71)	−2.51*** (0.42)	−2.18*** (0.41)
**Diet diversity**				
Total diet diversity score	3.49 (1.17)	4.21 (1.02)	−1.24*** (0.16)	−0.87*** (0.14)
*The number from on-farm production*	1.35 (0.71)	1.52 (0.7)	−0.44*** (0.07)	0.28** (0.13)
*The number from market purchase*	0.33 (0.64)	0.43 (0.74)	−0.11 (0.07)	−0.35*** (0.06)
Number of key food groups consumed in the past 24 h	1.07 (0.51)	1.09 (0.56)	−0.01 (0.05)	−0.10 (0.07)
*The number from on-farm production*	0.87 (0.45)	0.85 (0.46)	0.02 (0.05)	0.09 (0.07)
*The number from market purchase*	0.19 (0.44)	0.26 (0.51)	−0.08 (0.06)	−0.23*** (0.04)
Number of key food types consumed in the past 24 h	1.4 (0.81)	1.5 (0.87)	−0.10 (0.09)	−0.34*** (0.07)
*The number from on-farm production*	1.13 (0.75)	1.12 (0.76)	0.02 (0.09)	−0.02 (0.06)
*The number from market purchase*	0.2 (0.47)	0.28 (0.57)	−0.09 (0.06)	−0.25*** (0.05)
Number of key food groups consumed in the past 7 day	1.29 (0.62)	1.41 (0.65)	−0.14 (0.09)	−0.37*** (0.10)
*The number from on-farm production*	1.02 (0.52)	1.02 (0.53)	0.00 (0.06)	−0.04 (0.08)
*The number from market purchase*	0.3 (0.54)	0.41 (0.6)	−0.14* (0.07)	−0.32*** (0.06)
Number of key food types consumed in the past 7 day	2.21 (1.34)	2.61 (1.55)	−0.54** (0.19)	−1.01*** (0.15)
*The number from on-farm production*	1.74 (1.24)	1.96 (1.46)	−0.33* (0.17)	−0.46*** (0.14)
*The number from market purchase*	0.36 (0.68)	0.48 (0.74)	−0.14 (0.09)	−0.39*** (0.07)
**Food quantity**				
Amount of key food groups consumed in the past 24 h (g)	126.8 (139.6)	118.4 (99)	15.6 (12.2)	4.60 (14.9)
*The amount from on-farm production (g)*	105.1 (135.3)	87.7 (83.4)	26.6* (14.6)	31.7*** (16.4)
*The amount from market purchase (g)*	16.6 (46.5)	23.7 (59.4)	−8.5* (4.2)	−21.8*** (2.4)
**Crop diversity**				
Number of key food groups grown in the past 3 months	1.20 (0.55)	1.28 (0.61)	−0.14** (0.06)	−0.12 (0.09)
*Having any of the seed from self-saving source*	0.99 (0.57)	0.97 (0.68)	0.02 (0.06)	0.12 (0.10)
*Having any of the seed from market purchase*	0.55 (0.63)	0.80 (0.64)	−0.39*** (0.09)	−0.42*** (0.07)
Number of key food types grown in the past 3 months	2.61 (1.73)	3.00 (1.96)	−0.68** (0.28)	−0.39* (0.23)
*Seed from self-saving source*	1.73 (1.42)	1.68 (1.64)	0.02 (0.23)	0.36 (0.28)
*Seed from market purchase*	0.85 (1.16)	1.29 (1.32)	−0.69*** (0.15)	−0.72*** (0.16)

(1) Key food groups include Legumes, Dark Green Leafy vegetables, and Other Vitamin-A rich vegetables

(2) *p-value < 0.1; ** p-value < 0.05; *** p-value < 0.01.

Examining the diet diversity within the vegetable and legume food groups specifically, our baseline sample reports a consumption of 1.06 (SD = 0.51) out of three key food groups in the past 24 h (Table 4). This figure did not significantly increase when using the 7-day diet recall (Mean = 1.29, SD = 0.62). When investigating diet diversity at the vegetable types level, we found that respondents consumed only 2.21 (SD = 1.34) key types and predominantly from the Dark Green Leafy vegetable (DGLV) group (Table 4), which is rather commonly consumed in traditional Vietnamese diets across ethnic groups (Nguyen et al., 2023). However, the diversity within this food group was also low, with only 1.84 DGLV types consumed in the past 7 days. Even the Thai, who consumed most types from the key food groups, only consumed 2.07 types of DGLV in that recall period (Appendix 4-Figure 7). The diversity of other Vitamin A-rich vegetables and legume types was notably low across all respondents, averaging 0.22 legume type and 0.15 other Vitamin A-rich vegetables consumed in the past 7 days (Appendix 4- Fig. 6). Since we suspected that the low consumption of types from the other Vitamin A–rich vegetables and the legume food groups could be due to seasonal variation, we investigated the control group in two endline survey rounds. We found a greater presence of these two food groups in our respondents’ diets (Appendix 4- Fig. 7). However, despite this increase, the diversity of consumed types remained low, with the number of types averaging below 0.4 for the other Vitamin A-rich vegetables food group and below 0.5 for the legume food group consumed in the 7-day recall period (Appendix 4 - Fig. 7).

The low consumption of legumes and other Vitamin A-rich vegetables can be attributed to both culinary traditions and cultivation challenges. Among other Vitamin A–rich vegetables, pumpkins are the predominant choice, while other crops in this category, such as carrots and orange-fleshed sweet potatoes, are less common due to unsuitable growing conditions, and their limited role in traditional cuisine. Among legumes, soybeans are the most commonly consumed legume type. Although the H’Mong and Dao possess traditional legume landraces, they commonly consume either the young pods or leaves, which are nutritionally classified as vegetables. The consumption of dried and matured seeds, nutritionally categorized as `legumes’, is notably infrequent. These findings suggest that dietary habits and agricultural limitations are key barriers to increasing the consumption of these nutrient-rich foods. Addressing these challenges may require a behavioral change program that improves cultivation techniques and integrates these foods into regular diets.

The recorded dietary pattern in our sample was similar to the dominant diet in Northern Vietnam, particularly among the economically disadvantaged groups of the population, and this diet is characterized by high micronutrient deficiency risks (Nguyen et al., 2023). In a more comprehensive analysis focusing on female youths in the region, Tran et al. (2023) identified deficiencies in calcium, iron, Vitamin A, and Vitamin C. To mitigate these deficiencies, especially within our targeted group, it is beneficial to increase the diversity of DGLV and enhance the consumption of legumes and other Vitamin A-rich vegetables.

On-farm production emerged as the leading source for overall diet diversity, particularly for the three key vegetable and legume food groups. Our baseline sample sourced an average of 0.87 (SD = 0.45) groups, and 105 (SD = 135) grams of the key vegetable food groups from on-farm production in the past 24 h. In contrast, they obtained only 0.19 (SD = 0.44) groups, and 16.2 (SD = 46.5) grams from market purchases. This pattern persisted during the 7-day recall period (Table 4). A closer examination of individual food groups revealed that on-farm sourcing was especially crucial for DGLV and other Vitamin A-rich fruits and vegetables.

By contrast, the market significantly contributed to the provision of legumes (Appendix 4-Figure 7). Typically, market sources contributed nearly 10 times more than on-farm production to the legume consumption during baseline. Previous studies in Africa and Asia emphasized the complementary roles of on-farm and market in ensuring the accessibility of diverse foods (Nandi et al., 2021; Sibhatu et al., 2015). The market is typically important to the food that is too expensive to self-produce. Our findings that the market plays a major role in supplying legumes, while on-farm production is the key source of DGLV, are similar to what has been found for other countries across Africa and Asia (Bellon et al., 2023; Gupta et al., 2020; Nandi & Nedumaran, 2022). Unlike leafy vegetables, legumes are traded as dried grains, which can be transported much more easily which reduces the transportation cost for vendors.

Similarly, both self-saved and market-purchased seeds were pivotal in supporting crop diversity. Even though self-saving was the main seed source, the market played a crucial role as it accounted for approximately one third of the starting materials of the three key food groups (Appendix 4- Fig. 8). An in-depth analysis of individual food groups revealed that self-saving was the major source for the DGLV and other Vitamin A-rich vegetables food groups. The market contributed nearly 50% of the planting materials for the legume food group, and also around 30% of DGLV (Appendix 6-Figs. 7 and 8). As also reported for Africa and Mexico (Heindorf et al., 2021; Lipper et al., 2012), we thus find a significant role of local markets in enriching on-farm agrobiodiversity.

Additionally, our findings suggest the interaction of ethnicity and market access shaping the dietary outcomes. The Dao appeared to be the most subsistence-oriented ethnicity, as the majority of their diet diversity came from on-farm production. As the most isolated group, their consumption and cultivation of legumes, the food groups with the largest contributions of the market to both food consumption and planting materials – were the lowest. Among the three ethnicities, the Dao consumed 0.37 (SE = 0.10) less number of key food groups in the past 7 days compared to the Thai (Mean = 1.41, SD = 0.65) (Table 4). Despite living closest to the district market, the H’Mong exhibited similar diet diversity in key food groups to the Thai, suggesting that proximity to markets alone does not necessarily translate to higher diet diversity. In fact, their overall diet diversity score was the lowest among the three ethnicities, falling 1.24 (SE = 0.16) below that of the Thai.

These findings indicate that while market access plays a role in shaping diet diversity, its impact is mediated by ethnic-specific factors such as subsistence orientation, cultural dietary preferences, and economic constraints. The Dao’s limited engagement with markets restricts their diet diversity, whereas the H’Mong’s proximity to markets does not fully compensate for other barriers to diverse food consumption. This underscores the importance of considering both structural (market access) and sociocultural (ethnicity-related) determinants of dietary outcomes (Table 4).

### Balance check

Table 5 presented the imbalances between treatment and control groups of the baseline sample and disaggregated by ethnicities. Overall, the control and two treatment arms were balanced in terms of various household and respondent characteristics and outcome indicators (Table 5). However, some statistically significant imbalances were detected, especially among the training and control groups. The training group had 0.30 (SE = 0.16) more household members and lived 0.67 h closer to the district market than the control group. Moreover, this group significantly deviated from the control group in the following outcome variables at baseline. They consumed 0.19 (SE = 0.07) and 47.5 g (SE = 13.8) more key food types from on-farm production based on the 24 h recall data than the control group (Table 5).

Additionally, we noted imbalances across different treatment arms in the Thai and H’Mong ethnic subgroups. Compared to the control, the Thai training group lived closer to the district market, they consumed 0.16 fewer key food groups 0.19 fewer key food types from market purchase, and 0.20 more key food types from on-farm production. The seed provision group showed substantial differences from the control group in education level and age of respondents, age of household head, and also reported higher consumption of key food groups, particularly from on-farm sources. The H’Mong training group differed from the control group in household characteristics and diet outcomes. Disparities were also noted between seed provision and control groups, especially in the quantity and number of crop types from key food groups cultivated (Table 5). The full balance tables of baseline and endline sample can be found in Appendix 5 – Tables 4 and 5.

We consider that the observed imbalances did not lead to biased estimates in the approaches controlling for both household and respondent characteristics and baseline outcomes (models 1 and 3, the ANCOVA models). Treatment effects of diet diversity measured at 24 h derived from model 2 should be considered carefully since this model does not control for baseline outcomes. Below, we present the results of our preferred model, model 1. The results for the other models are presented in Appendix 5- Tables 6 and 7.

**Table 5:** Mean (SD) at baseline of household and respondent characteristics and outcome variables of the control groups in the total baseline sample, and Thai and H’mong baseline samples separately; regressions coefficients (Robust SE) for treatment assignments. Only imbalanced variables shown

Dependent variables	Total sample			Thai sample			H’Mong sample		
	Control (*N* = 374)	Training (*N* = 596)	Seed provision on top of the training (*N* = 295)	Control (*N* = 90)	Training (*N* = 127)	Seed provision on top of the training (*N* = 64)	Control (*N* = 91)	Training (*N* = 200)	Seed provision on top of the training (*N* = 97)
	Mean (SD)	Coefficients (SE)	Coefficients (SE)	Mean (SD)	Coefficients (SE)	Coefficients (SE)	Mean (SD)	Coefficients (SE)	Coefficients (SE)
**Household and respondent characteristics**									
Household head is female								0.06* (0.03)	−0.03** (0.02)
Household head completed primary school or higher								−0.13** (0.06)	
Respondent completed primary school or higher						0.12* (0.06)			
Age of Household head						−2.70* (1.3)			
Age of respondents						−3.40* (1.8)			
Number of household members		0.30* (0.16)						0.42** (0.16)	
Walking distance to nearest district market (hours)		−0.67* (0.38)			−0.62* (0.31)				
Walking distance to nearest fresh food market (hours)						−0.06* (0.03)			
**Outcome indicators**									
***Diet diversity***									
The number of key food groups consumed in the past 24 h from market purchase					−0.16* (0.08)				
Number of key food types consumed in the past 24 h								0.24* (0.13)	
Number of key food types consumed in the past 24 h from on-farm production		0.19*** (0.07)						0.24** (0.10)	
***Food quantity***									
Amount of key food groups consumed in the past 24 h (g)		43.2*** (12.4)				35.1* (17.8)		61.7*** (16.1)	−46.6*** (16.6)
The amount of key food groups consumed in the past 24 h from on-farm production (g)		47.5*** (13.8)			24.5* (12.1)	18.8** (7.4)		60.8*** (20.0)	−40.3*** (17.9)
***Crop diversity***									
Number of key types grown in the past 3 months with seeds from self-saving source			0.33** (0.16)	1.67 (1.59)	−0.17 (0.26)				

(1) Key food groups include Legumes, Dark Green Leafy vegetables, and Vitamin-A rich vegetables

(2) *p-value < 0.1; ** *p*-value < 0.05; *** *p*-value < 0.01

(3) The sample size in the parentheses are number of observations

### Effects of NSA interventions

#### Effects of training on nutritional knowledge

Table 6 presented the treatment effects estimated by the ANCOVA model using baseline sample (model 1). Our analysis shows a statistically significant increase in participants’ nutritional knowledge due to the training. Specifically, we observed an improvement of 1.04 points (SE = 0.41) compared to the control (Mean = 2.63, SD = 4.50) estimated by our baseline sample (Table 6). Notably, the improvement of nutrition knowledge was particularly significant for the H’Mong, unlike the Thai, where no such improvement was noted (Table 6). This disparity may stem from H’Mong’s lower baseline knowledge level, which inherently allowed for a greater potential for improvement. Further analysis of Local Average treatment effects (LATEs) reinforces the sub-group differences, showing a positive correlation between treatment engagement and enhanced nutrition knowledge exclusively for the H’Mong. No significant treatment effects were found for the Thai across all levels of treatment engagement (Appendix 6- Fig. 9). H’Mong participants who joined at least one training session showed a significant knowledge increase of 1.2 points (90% Confidence Intervals = 0.9 to 1.6), and those who joined at least three training sessions obtained 1.4 points (90% Confidence Intervals = 1.0 to 1.8) higher compared to the control group (Mean = 0.5, SD = 1.8). Despite these improvements being notable in magnitude, the final level achieved and the practical implications of the training remained insubstantial, given that the maximum achievable score is 20 (Appendix 6- Fig. 9).

Even though the quantitative treatment effects varied by sub-group, our qualitative findings exhibited common factors hindering knowledge attainment across all ethnicities. First, the participants found nutrition terms and concepts complex. Our H’Mong and Dao facilitators and enumerators faced difficulties in explaining and translating the word `nutrition’, due to a lack of equivalent terms in their languages. One member in Sa Pa shared *“The most difficult things [in the training curriculum] for the people here is nutrient classification*,* how to consume enough iron*,* and vitamins. They find it hard to understand and to learn because they are illiterate”* . However, literacy alone does not fully explain the difficulties participants faced. Even among those with higher proficiency in Vietnamese, such as the Thai group, challenges persisted: *“The sessions were difficult to understand*” *[FGD 7, Thai Mai Son, Training beneficiaries]*. This suggests that while illiteracy may have been a contributing factor for some participants, the complexity of the concepts was a major hurdle as participants are unfamiliar with nutritional classifications.

The research team made significant efforts to make the training relevant and understandable for the target group. We hired local trainers and trained them in two Training of Trainers workshops. After discussion with the trainers, we decided to simplify the training layout, provide audio podcasts in local languages, and offer constant assistance for the local trainers by the field assistants, primarily onsite, with occasional online assistance when the COVID-19 pandemic made in-person meetings difficult. We also attempted to tailor the training to respondents’ requests by developing new material in response to participants’ requests. However, the results indicate that these efforts were inadequate. The key challenges are the insufficient project duration, the mismatch between training methods and participants’ preferred learning styles, and disrupted training schedules. The hurdles introduced by the COVID-19 pandemic exacerbated the situation.

Limited project duration posed two fundamental challenges. First, limited project duration hindered the effective delivery of intended knowledge to the targeted group. A longer project duration not only would have allowed the implementation of actions that accommodate the unforeseen disruptions from the pandemic but also given space for information reinforcement. One of the trainers expressed: *“Maybe [the project team] should organize more meetings and training*,* so that villagers would understand better the benefits of the project”* *[KII5, H’Mong Sa Pa, Facilitators]*, or a training participant noted, “*[I] want they come back and train us more if they have time” [FGD4, H’Mong Mai Son, Training beneficiaries]*. These feedbacks reflect the need for more prolonged and intensive training to realize and sustain any knowledge improvement. Longer training period and higher training intensity also give greater opportunities and flexibility to farmers to participate into the training, thus increase the participation rate.

Second, insufficient duration prevented the research team from developing necessary skills to effectively adapt content to the target audience and bridge the gap between the research team and participants, especially when they come from different backgrounds, as in our case. Beyond technical knowledge, trainers required training in communication techniques, participant engagement, and adaptive teaching methods. Without this preparation, maintaining a strong connection between the research team and participants proved challenging (Di Prima et al., 2022).

Additionally, while simplifying the training layout improved knowledge accessibility, it also reduced interactive and participatory elements, which are critical for effective learning (Kadiyala et al., 2018). The initial training design included four practical sessions out of eight sessions in total, with two participatory sessions on cooking and two on agriculture. However, some participants believed that the number of practical sessions provided was not sufficient, and they stated more practical sessions should be added. A Thai club member shared “*I wanted to have a [more] hands-on experience because I am illiterate*” [FGD7, Thai Mai Son, Training beneficiary]. *“[The theoretical sessions] were easy to understand, but [I] forgot them very fast, [because I] only listen without practice*” *[FGD 5, H’Mong Mai Son, Training beneficiary]*.

Observations from field assistants reinforced this need for more experiential learning. One assistant explained that farmers used `illiteracy’ to refer to their difficulty in adhering to classroom-based training. In his field report, he wrote “*I have explained many times and found ways to explain the contents most easily, but the farmers were not familiar with studying so they were sleepy, they found it hard to concentrate and to understand the knowledge*”. As farmers, they excel in learning through hands-on experience, highlighting the need for more practical training sessions to enhance knowledge comprehension and retention. Additionally, the COVID-19 pandemic further complicated the situation. While online assistance was provided, it lacked the interaction of in-person support, limiting engagement and mutual responsiveness. The logistical challenges of remote facilitation also prevented conducting some practical agriculture sessions, reducing opportunities for hands-on learning and personal interaction.

Furthermore, the disrupted training schedule, partly due to the COVID-19, made the knowledge retention more challenging. As noted by a participant “*The training must be at least once per month, people will* *forget everything if [the training gap] is longer than that*” *[FGD4, H’Mong Mai Son, Training beneficiary]*. A local trainer echoed this concern “*The training should be more frequent. Farmers will be less motivated to participate [to the next session] if [the sessions] is too far apart*” *[KII1, Thai Mai Son, Facilitators]*.

**Table 6:** Intent to treat (ITT) treatment effects (Robust SE) of nutrition training alone and seed provision on top of the training on outcome variables estimated by model 1 using ANCOVA estimator for the total sample and the Thai and H’mong samples separately: using the baseline sample with correction for baseline outcomes

Dependent Variables	Total sample			Thai sample			H’Mong sample		
	Control (Mean(SD))	Training	Seed provision on top of training	Control (Mean (SD))	Training	Seed provision on top of training	Control (Mean (SD))	Training	Seed provision on top of training
**Nutrition knowledge score**	2.63 (4.50)	1.04** (0.41)	0.33 (0.33)	4.79 (5.64)	1.02 (0.78)	0.55 (0.67)	0.59 (1.79)	1.11*** (0.18)	0.43 (0.31)
**Diet diversity**									
Number of key food groups consumed in the past 24 h	1.11 (0.64)	0.09* (0.05)	0.01 (0.05)	1.22 (0.69)	0.16** (0.05)	−0.03 (0.07)	1.06 (0.57)	0.00 (0.07)	−0.01 (0.06)
*The number from on-farm production*	0.81 (0.56)	0.07 (0.06)	0.01 (0.05)	0.76 (0.56)	0.28*** (0.07)	−0.05 (0.06)	0.84 (0.56)	−0.10 (0.07)	0.00 (0.05)
*The number from market purchase*	0.32 (0.54)	−0.03 (0.05)	0.01 (0.04)	0.5 (0.63)	−0.16** (0.07)	0.02 (0.06)	0.20 (0.45)	0.05 (0.07)	−0.02 (0.04)
Number of key food types consumed in the past 24 h	1.45 (0.97)	0.16 (0.1)	0.04 (0.07)	1.71 (1.07)	0.29* (0.14)	0.03 (0.04)	1.34 (0.87)	−0.08 (0.15)	0.01 (0.09)
*The number from on-farm production*	1.06 (0.86)	0.14 (0.1)	0.04 (0.07)	1.15 (1.01)	0.42** (0.16)	0.00 (0.11)	1.03 (0.78)	−0.19 (0.13)	0.05 (0.08)
*The number from market purchase*	0.32 (0.56)	−0.03 (0.05)	0.01 (0.04)	0.5 (0.65)	−0.15* (0.08)	0.02 (0.06)	0.21 (0.47)	0.04 (0.07)	0.00 (0.04)
Number of key food groups consumed in the past 7 day	1.71 (0.74)	−0.01 (0.06)	0.10** (0.04)	1.96 (0.70)	−0.07 (0.10)	0.13** (0.06)	1.54 (0.76)	0.03 (0.07)	0.01 (0.05)
*The number from on-farm production*	1.15 (0.58)	0.03 (0.05)	0.08** (0.04)	1.20 (0.60)	0.14 (0.08)	0.05 (0.05)	1.06 (0.6)	−0.04 (0.05)	0.05 (0.04)
*The number from market purchase*	0.68 (0.74)	−0.08 (0.07)	0.03 (0.05)	0.93 (0.72)	−0.28** (0.11)	0.12* (0.06)	0.58 (0.77)	0.03 (0.08)	−0.07 (0.07)
Number of key food types consumed in the past 7 day	2.92 (1.73)	0.07 (0.17)	0.20* (0.10)	3.94 (1.73)	−0.01 (0.29)	0.37* (0.21)	2.34 (1.4)	−0.09 (0.16)	0.05 (0.08)
*The number from on-farm production*	2.06 (1.45)	0.18 (0.16)	0.07 (0.09)	2.78 (1.66)	0.38 (0.29)	0.05 (0.21)	1.56 (1.07)	−0.14 (0.16)	0.06 (0.1)
*The number from market purchase*	0.75 (0.88)	−0.13 (0.08)	0.06 (0.06)	1.03 (0.89)	−0.36** (0.13)	0.18** (0.08)	0.64 (0.89)	0.01 (0.10)	−0.06 (0.09)
**Food quantity**									
Amount of key food groups consumed in the past 24 h (g)	202.1 (229.2)	−14.5 (19.5)	19.9 (14.7)	212.0 (248.6)	−4.5 (33.4)	8.9 (25.8)	232.4 (232.2)	−36.93 (23.9)	34.9 (25.9)
*The amount from on-farm production* *(g)*	152.0 (197.4)	−4.3 (17.1)	4.2 (11.7)	141.9 (190.8)	27.3 (31.9)	−8.6 (26.9)	186.2 (222.9)	−45.8*** (15.3)	13.9 (9.8)
*The amount from market purchase* *(g)*	39.65 (128.8)	−16.6* (8.4)	16.8 (12.6)	63.53 (181.32)	−36.2*** (10.4)	14.2 (10.9)	28.5 (74.0)	−1.9 (10.3)	25.9 (25.1)
**Crop diversity**									
Number of key food groups grown in the past 3 months	2.13 (0.79)	−0.14* (0.08)	0.13** (0.05)	2.24 (0.74)	−0.11 (0.12)	0.09 (0.10)	2.05 (0.86)	−0.08 (0.08)	0.16** (0.06)
*Having any of the seed from self-saved source*	1.84 (0.82)	−0.12 (0.09)	−0.02 (0.05)	1.78 (0.79)	0.01 (0.14)	−0.11 (0.09)	1.83 (0.87)	−0.13 (0.08)	0.00 (0.08)
*Having any of the seed from market purchase*	0.78 (0.83)	−0.04 (0.08)	−0.02 (0.08)	1.21 (0.81)	−0.15 (0.16)	−0.06 (0.21)	0.56 (0.77)	−0.01 (0.09)	0.03 (0.06)
Number of key types grown in the past 3 months	5.28 (3.46)	−0.41 (0.42)	0.34* (0.17)	7.32 (4.12)	−0.67 (0.84)	0.08 (0.27)	3.73 (1.81)	−0.44** (0.17)	0.60*** (0.15)
*Seed from self-saving source*	3.98 (2.53)	−0.37 (0.33)	−0.13 (0.14)	5.06 (3.02)	−0.26 (0.69)	−0.27 (0.29)	2.97 (1.72)	−0.45*** (0.15)	−0.04 (0.10)
*Seed from market purchase*	1.15 (1.67)	−0.04 (0.16)	−0.10 (0.13)	1.98 (2.09)	−0.32 (0.31)	−0.34 (0.29)	0.66 (0.96)	0.01 (0.14)	0.08 (0.12)
**Model specification**									
N		973			376			463	
Control for household and respondents characteristics		Yes			Yes			Yes	
Control for baseline outcomes		Yes			Yes			Yes	
Control for missing baseline dummy		No			No			No	

(1) Coefficients, Robust standard errors and p-values were retrieved from the model $\:\:{Y}_{ij}^{1}=\alpha\:+{\beta\:}_{1}{T}_{1j}+{\beta\:}_{2}{T}_{2j}+\:{\rho\:}_{1}{X}_{ij}+\:{\rho\:}_{2\:}{{MA}_{ij}+\:\epsilon\:}_{ij}\left(2\right)$, using pooled sample of two endlines, where $\:{T}_{1}$ is a treatment dummy for receiving training, and $\:{T}_{2}$ is a dummy for receiving seeds on top of the training.

(2) (*) denotes model *p*-value * *p*-value < 0.1; ** *p*-value < 0.05; *** *p*-value < 0.01.

(3) Coefficients in <b>bold</b> indicates those with corrected p-value from multiple hypothesis testing less than 0.1. The p-values were retrieved from Romano-Wolf procedure, which controls the family wise error rate (FWER) for three families: diet diversity, food quantity and crop diversity

#### Effects of the training intervention on diet and crop diversity

We found a limited overall effect of the training on diet and crop diversity across the entire sample, with an exception of a negative treatment effect on diet diversity from market purchases, even though this effect is no longer significant after multiple hypothesis testing (Table 6). This result was consistent across all estimation approaches (Table 6 and Appendix 5 – Tables 6 and 7). Subgroup analysis suggests a shift from reliance on market purchases to greater use of on-farm production for diet diversity. This shift was particularly pronounced among the Thai. The training reduced the number of key food groups (Coefficient=−0.28, SE = 0.11) consumed in the past 7-day from market purchase, and increased the number of key food groups consumed in the past 24 h from on-farm production (Coefficients = 0.28, SE = 0.07). The positive effect on on-farm sources remained significant after multiple hypothesis testing (Table 6). These results were also recorded using different estimation models (Appendix 5- Tables 6 and 7). Credible Interval obtained from the Bayesian framework (Sect. 3.3.1) pointed to a similar direction as the confidence intervals estimated from the ANCOVA model (Appendix 7 – Fig. 11). One potential mechanism of this effect among the Thai could be the interaction between the beneficiaries’ self-interpretation of the training and the impact of the COVID-19 pandemic. Specifically, the Thai training group might have interpreted the purpose of the training as advocating for diet diversity exclusively from on-farm production, as reported by Thai training beneficiaries, such as “Q: *How did your vegetable purchase change before and after the training? *A1: *[I buy] less [vegetables] because I can save my own seeds, so I can grow my own vegetables.* A2: *The [vegetable purchase] frequency is less because I can grow my own vegetables*” *[FGD 7, Thai Mai Son, Training beneficiaries]*. The market disruptions caused by the pandemic could have reinforced this intention, leading to a reduction in the diversity and quantity of vegetables and legumes purchased from the market.

Notably, despite of the observed shift in diet, we did not find an increase in crop diversity among the Thai (Table 6). This could be due to the fact crop diversity was already relatively high among the Thai, compared to the H’Mong and Dao group (Table 4), leaving little room for improvement. However, the increase in diet diversity without a corresponding rise in crop diversity may suggest that the existing level of crop diversity among the Thai was already sufficient to support improvements in their diet. This, though, cannot be checked from our data.

Nevertheless, these shifts did not necessarily improve total diet diversity. While the Thai training participants displayed a higher net total effect on diet diversity than the control group according to the 24-hour diet recall data, we did not observe a similar effect with the 7-day recall data (Table 6). During the COVID-19 pandemic, on-farm production was the reliable food source to ensure a diet quality for farmers (Connors et al., 2021). This suggests that among the Thai, on-farm production cannot replace completely market purchases. Therefore, to improve diet diversity, the promotion of both food sources are necessary.

Additionally, our results indicate the training group grew 0.14 (SE = 0.08) less key food groups in the past three months than the control group (Table 6). This effect remained significant after multiple hypothesis testing correction and estimation using two other estimation approaches suggesting a similar negative effect of the training (Table 6; and Appendix 5- Tables 6 and 7). Furthermore, we found that the reduced crop diversity was primarily driven by the H’Mong, and typically for crops grown from self-saved seed sources (Table 6).

However, we cannot explain the underlying mechanism for this effect. We suspect that the observed effects could be due to a decrease in crops grown for sale, in response to disrupted market access due to the COVID-19 pandemic. The training could amplify this tendency since the group setting offered women a platform to discuss issues relevant to their lives (Nichols, 2021), which included the development of the COVID-19 pandemic. This forum did not just help farmers to be better informed, but could also have reinforced bias, as part of the social learning process (Stone, 2016). To test this hypothesis, we investigated the treatment effects on output market participation and the diversity of crops for sale. However, the auxiliary analysis’s results did not support our auxiliary hypothesis, as we found no treatment effects on the two investigated variables (Appendix 6- Fig. 10).

#### Effects of seed provision on top of the training

Our study found that seed provision when combined with nutrition and agriculture training significantly contributed to several treatment effects in the total sample. Unsurprisingly, the treatment helped to increase the crop diversity of the seed recipients. We found that the seed provision led to 0.13 (SE = 0.05) more key food groups, and 0.34 (SE = 0.17) more key food types being cultivated compared to non-seed recipients in the training group (Table 6). The treatment effects remain significant after multiple hypothesis testing. These findings were consistent across different estimation methods (Appendix 5- Tables 6 and 7), and when using the Bayesian approach (Appendix 7 – Fig. 12). The enhanced crop diversity was translated into a more diverse diet; as we found seed provision increased the number of key food groups consumed by 0.10 (SE = 0.04), compared to the training only group, mainly from on-farm production (Coefficients 0.08, SE = 0.04). Our finding is in line with other studies during the pre-COVID period (Daidone et al., 2017).

Although the effect size on diet is modest, it is meaningful given the limited impact of training alone. Considering its low operational cost, seed provision serves as a cost-effective complement to the training, offering additional pathways to improve dietary outcomes. While the total effects of the intervention bundle may not be significant, differentiating the impact of each treatment provides a clearer understanding of how our NSA functioned.

Heterogenous treatment effects analysis uncovers intriguing mechanisms behind the observed outcomes in the total sample. Improved diet diversity was driven by the Thai, while increased crop diversity was attributable to the H’Mong seed recipients. The different treatment effects of seed provision in the two sub-samples could be explained by the varying timing of seed distribution in the two study regions, which was determined by the travel restrictions policy in response to the COVID-19 pandemic. In Sa Pa, where the H’Mong is the majority, seed recipients received their last batch of seed three months before the first endline. In Maison, home to the Thai community, this period was six months (Appendix 1- Table 1). Growth duration for distributed crops was typically around two to three months.

The lack of treatment effects on crops from purchased and self-saved seeds among the H’Mong suggests that the improved crop diversity was due to the provided seeds. This is in line with the recorded seed distribution agenda. The missing treatment effect among the Thai raises questions about the long-term sustainability of the seed provision. Nevertheless, it should be noted that the Thai’s crop diversity had a smaller margin for improvement, as their crop diversity at baseline was relatively high compared to the other two ethnicities (Table 4).

Despite the limited effect of seed provision on crop diversity among the Thai, the positive treatment effect of seed provision on diet diversity suggests another mechanism by which seed provision could enhance diet diversity. When integrated into nutrition training, seed provision could serve as a practical reinforcement of the training’s dietary advice, and underscore the necessity and options for diet diversity. Consequently, it motivates them to be more engaged in the interventions and pursue the recommended diet.

On the other hand, even though the H’Mong experienced improved crop diversity, this did not translate into enhanced diet diversity. Farmers in Sa Pa reported several issues relating to the provided seeds, including poor germination rates and low yields, as the consequences of the disrupted seed distribution schedule due to the COVID-19 pandemic. One seed recipient noted, *“The beans did not give pods”[FGD 12- H’Mong Sa Pa*,* seed recipients]*. The poor harvest of beans was likely due to seasonality, as September was too late for bean planting in Sa Pa (Appendix 1 – Table 1). To address this issue, the research team distributed Faba beans, which are more cold-resistant. However, their taste failed farmers’ expectations, “*The Faba bean grains are too hard*,* and their taste is not good” [FGD 10 – H’Mong Sa Pa*,* seed recipients].* Additionally, unfavourable weather could be a contributing factor to poor treatment effects on diet. The field assistant reported unusually high rainfall levels in October and November, which was detrimental for young plants, which also affected DGLVs.

These findings highlight the challenges and uncertainties associated with seed provision interventions aimed at improving diet diversity via agrobiodiversity. There is no doubt that farmers possess a wealth of agricultural knowledge and are experts in dealing with local conditions (Almekinders et al., 2021; Lambrou & Laub, 2007). However, when implementing interventions to enrich the current agrobiodiversity, the local knowledge system may be insufficient in dealing with novel or under-cultivated crops. This is even more relevant in the context of climate change when weather patterns become increasingly unpredictable.

Our results show that providing agriculture training and individual assistance from unexperienced field assistance alone is not sufficient. It is crucial to integrate robust extension services into seed provision intervention. These services not only help farmers address challenges related to cultivating novel crops but also guide them in selecting appropriate crops and cultivars for their specific local conditions. Moreover, the extension staffs should be trained to be nutrition-sensitive, enabling them to offer advice that is both agriculturally and nutritionally beneficial to farmers.

Together with the findings of the training’s impact on diet, our results also suggest that NSAs aiming to enhance diet quality via several impact pathways should establish an effective monitoring. Such system would allow for timely updates and adjustments to interventions based on local needs. It should involve a local support network, where each member plays a distinct role and leverages specific capacities aligned with each pathway. Building and strengthening the capacity of this team is crucial, underscoring the importance of local capacity-building programs (Asare-Nuamah et al., 2019; Di Prima et al., 2022).

## Conclusion

Our study explores the vegetable and legume consumption and cultivation practices among ethnic minorities in northern Vietnam. Additionally, we inspected the impacts of an NSA which included nutrition training and seed provision on top of that training to part of the study groups during the COVID-19 pandemic. We found that the targeted groups, especially H’Mong and Dao, experienced a notable lack of diet diversity over vegetable and legume food groups. Even though in general, ethnic minorities had a good access to agrobiodiversity, this diversity was not evenly distributed across food groups and ethnicities, due to various factors, including culinary culture and cultivation limitations. Our baseline findings also highlight the vital roles of food from both market purchase and on-farm production in enhancing diet diversity. Similarly, self-saved seeds and market-purchased seeds complementarily harness crop diversity.

Our findings point out various contextual factors leading to the success and failure of an NSA. This does not only include the initial conditions, but also other developments along the implementation. Insufficient project duration, the mismatch between training methods and participants’ preferred learning styles, and disrupted training schedules, which were exacerbated by the COVID-19 pandemic, played a crucial role. These factors interact further with existing local conditions, leading to varying treatment effects across ethnicities.

To strengthen the NSAs design, future projects should evaluate the readiness of target communities for such interventions. This includes assessing how NSAs align with local interests, and potential to shifts in consumption and crop production. Considerations should include existing culinary culture, farming practices, market access, and openness to innovation. Additionally, practitioners should examine the alignment between training concepts and local worldviews, participants’ learning preferences, farming calendars, the strength of local support systems, including trainers and feedback mechanisms. These insights will help to determine feasible implementation durations, training materials, layout and frequency.

Furthermore, in the context of a strong culture of vegetable cultivation and consumption and limited market access, adding seed provision could enhance the probability of success. This intervention not only expands current agrobiodiversity, which is then translated into a better diet but also reinforces the advice of diet diversity, especially when being offered in the context of nutrition training. To increase the chance of success of this intervention, providing extension service support is advisable, particularly in the context of increasingly unpredictable weather patterns. The extension staff should also be trained in nutrition sensitive agriculture’s concepts and logic, to be able to give nutrition-oriented advices to farmers.

## Supplementary Information

Below is the link to the electronic supplementary material.

Supplementary Material 1

ESM 2 (DOCX 1.04 MB)

## Footnotes

1 The original name of the food group in FAO classification is `other Vitamin-A rich fruits and vegetables’. However, as this study focusses on vegetables, we decided to drop the fruits and renamed the food group to `other Vitamin A-rich vegetables’.

2 According to FAO (2021), the ‘Pulses food group’ includes dried, matured seeds of beans, peas and lentils, meanwhile ‘legumes’ refers to plants from the *Leguminosae* family. To simplify the terminology, in this study, we use the `Legume’ food group as term instead of Pulses, and `Legume crop’ to refer to the plants which produce these legume seeds.

3 The term vegetable type is used as some vegetable species (e.g. *Brassica oleracea*) host rather different vegetables, like broccoli and white cabbage, consumers would not consider from a single species while some vegetables (e.g. pumpkins) could come from more than one species but would be considered as a single vegetable. With the term vegetable type the paper follows the more common distinction made by consumers’ and nutritionists rather than the botanical one.

4 Check our Pre-Analysis Plan for further details on power analysis https://osf.io/9qemb.

5 The `Food Atlas’ includes only a selected set of the most commonly consumed food items. For items not available in the atlas, we used the closest matching items as a reference for measurement. For example, mustard greens were used to estimate the quantity of H’Mong mustard, or chayote (slices) were used to substitute for kohlrabi (slices).

6 The Pre-Analysis Plan of this study can be found at https://osf.io/9qemb.

7 The regression models used for the multiple imputations included the missing variables, individual and households characteristics.
