# Supplementary material for: Do nutrition-sensitive agriculture interventions work among ethnic minorities in Northern Vietnam amidst the COVID-19 crisis?
Source: Food Secur. 2025 Sep 4;17(5):1153–74. doi: 10.1007/s12571-025-01580-2 (PMC12549742; doi:10.1007/s12571-025-01580-2)
Supplement: Supplementary file 1 — Supplementary Material 1 [file 12571_2025_1580_MOESM1_ESM.docx]

**Title: Do Nutrition-Sensitive Agriculture Interventions (NSAs) work among ethnic minorities in Northern Vietnam amidst the COVID-19 crisis?**

**Lan Thuy T. Nguyen ^a,b,c,^^[[1]](#footnote-1)^, Marrit van den Berg^a,1^, TjeerdJan Stomph^c^, Deborah Nabuuma^b^**

**^a^** Development Economics Group, Wageningen University and Research, the Netherlands

**^b^**  Bioversity International, Penang, Malaysia

^c^ Crop Science Analysis, Wageningen University and Research

**Abstract**


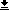

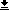
Undernutrition remains a significant challenge among ethnic minorities in Northern Vietnam, possibly due to limited diet diversity. Our study explored the potential of nutrition-sensitive agriculture (NSA) interventions to improve diet quality among the Thai, H’Mong, and Dao communities using a mixed-methods approach. Conducted between December 2020 and July 2022 during the COVID-19 pandemic, the study involved 600 households across 36 clusters, divided into two treatment groups: one received nutrition and agricultural training, another received this training plus a variety of vegetable seeds, and a control group without any intervention. We focused on dietary and crop diversity, especially in vegetables and legumes. Quantitative data was collected through one baseline and two end-line rounds to ensure sufficient statistical power, while qualitative data included 14 focus group discussions, seven in-depth interviews, and field notes from field assistants. Our findings revealed that dietary diversity, particularly in vegetables and legumes, was limited among the H’Mong and Dao, underscoring the potential of NSAs to improve their diet. Market access and on-farm production were found to complement each other in enhancing both dietary and crop diversity across all three communities. The impact of the NSA intervention highlighted the complexity of contextual factors, including initial conditions and the ongoing effects of the COVID-19 pandemic, which influenced the outcomes in unpredictable ways. Nonetheless, combining seed provision with nutrition and agricultural training emerged as a promising strategy to enhance both diet and crop diversity, particularly given the strong vegetable cultivation practices and limited market access in these communities.

.

**Keywords*:*** Diet diversity, impact pathways, agrobiodiversity, impact evaluation, social equity.

**Statements and Declarations**

**Authorship contributions**

Lan Thuy T. Nguyen**:** conceptualization, methodology, data curation, software, formal analysis, writing-original draft, writing-review and editing; Marrit van den Berg: conceptualization, methodology, writing-review and editing, supervision; TjeerdJan Stomph: conceptualization, methodology, writing-review and editing, supervision, funding acquisition; Deborah Nabuuma: conceptualization, methodology, writing-review and editing, project administration.

**Acknowledgement**

The author would like to thank the funders for making this research possible. We express our great gratitude toward Mr. Hoang The Ky from Bioversity International, Vietnam, Ms. Pham Thi Mai Huong from CIAT, Vietnam, and the research team in Fruit and Vegetable Research Institute, Vietnam for supporting the fieldwork and data collection. We are indebted to the Women’s Union and Extension department in Sa Pa and Mai Son districts, the enumerators, field assistants, and study participants.

**Fundings**

This study was part of a project within the seed system development program component of the Netherlands- CGIAR research program, project number W.08.240.101, which was funded by the Dutch Research Council (NWO). This work was also carried out under the CGIAR Research Initiatives on Sustainable Healthy Diets through Food systems Transformation (SHiFT).

**Employment**

Lan Thuy T. Nguyen: Wageningen University and Research & Bioversity International; Marrit van den Berg: Wageningen University and Research; TjeerdJan Stomph: Wageningen University and Research; Deborah Nabuuma: Bioversity International.

**Financial Interests**

The authors declared that they have no financial interests with any company.

**Non-financial Interests**

The authors declare they have no non-financial interests with any organization.

1. Corresponding author at Development Economics Group, Wageningen University and Research, Hollandseweg 1, 6706 KN Wageningen, the Netherlands

   Email address: [lan.nguyen@wur.nl](mailto:lan.nguyen@wur.nl) (Lan Thuy T.Ngyen); [marrit.vandenberg@wur.nl](mailto:marrit.vandenberg@wur.nl) (Marrit van den Berg). [↑](#footnote-ref-1)
