# Supplementary material for: Do nutrition-sensitive agriculture interventions work among ethnic minorities in Northern Vietnam amidst the COVID-19 crisis?
Source: Food Secur. 2025 Sep 4;17(5):1153–74. doi: 10.1007/s12571-025-01580-2 (PMC12549742; doi:10.1007/s12571-025-01580-2)

## Appendix 1: Implementation process and compliance rate

**Table 1:** Implementation timeline of seed provision and provided seeds

| Studied sites | Time | Cultivar distributed |
| --- | --- | --- |
| Mai Son | April 2021 | Spineless Amaranth, Ceylon Spinach, Pumpkin (for leaves), Pumpkin (for fruits), French bean |
|  | September 2021 | Chrysanthemum, Carrot, Chinese Mustard, Faba bean |
| Sa Pa | September 2021 | Chinese mustard, Gai Lan, Mustard Green (Choy sum), French bean, Black bean, Mung bean, rice bean |
|  | November 2021 | Faba bean |

**Table 2:** Participation rates of training and seed provision interventions from endline sample

|  | Baseline sample | | | | Endline sample | | | |
| --- | --- | --- | --- | --- | --- | --- | --- | --- |
|  | Total sample | Thai | H’Mong | Total sample | | Thai | H’Mong |  |
| Eligible to join the training | 368 | 127 | 200 | 426 | | 140 | 290 |  |
| Join at least 01 training session | 82% | 89% | 79% | 73% | | 83% | 73% |  |
| Join at least 03 training session | 71% | 76% | 71% | 63% | | 71% | 66% |  |
| Joining all session | 36% | 38% | 40% | 32% | | 35% | 39% |  |

Appendix 2: Sample calculation for baseline survey and randomization design

Since we had a little room to improve the number of clusters, our task was geared towards finding the optimal cluster size. We computed the Minimum Detectable Effect size (MDE) with the variations of cluster size using this formula:

$$MDE=t_{1-k}+t_{\alpha/2}\sqrt{\frac{1}{P(1-P)}\times\frac{\sigma^{2}}{N}\times(1+(m-1)\times ICC)}$$

In which:

$t_{1-\propto/2}$: Critical value of a two-tailed test at significant level $\propto=10\%$

$t_{1-k}$ : Critical value of a two-tailed test with k is number of clusters, k=18

m: Average number of units per clusters

$N$: Total sample size

$ICC$: Intra cluster correlation

$\sigma^{2}$: Standard deviation

P : the proportion of the sample assigned to the treatment arms. In our study, P=0.5

Other parameters were computed from the survey of 409 households in the same study regions conducted by the Alliance of Bioversity and CIAT in 2019 (Table 3).

**Table 3:** Descriptive statistics of several outcome variables

| **Indicator descriptions** | **Indicators name** | **Mean** | **Standard deviation**$\mathbf{(}\boldsymbol{\sigma}^{\mathbf{2}}\mathbf{)}$ | **Intra cluster correlation (ICC)** |
| --- | --- | --- | --- | --- |
| **Diet diversity** |  |  |  |  |
| Diet diversity score (MDD-W) | MDD-W | 3.6 | 1.06 | 0.04 |
| Number of key food groups consumed | Focused MDD | 1.6 | 0.75 | 0.06 |
| Number of Vitamin A rich vegetable types consumed in the past 24h | VitA rich species consumed | 0.13 | 0.3 | 0.001 |
| Number of key food types consumed in the past 24h | Focused species consumed | 2.3 | 1.4 | 0.13 |
| **Crop diversity** |  |  |  |  |
| Number of vegetable and legume types grown in the past 12 month | Crop grown | 5.3 | 3.5 | 0.57 |
| Number of key food groups grown in the past 12 months | Focused species grown | 2.68 | 1.5 | 0.2 |
| Number of Vitamin A rich vegetable types grown in the past 12 month | VitA rich species grown | 0.57 | 0.66 | 0.39 |

Figure 2 illustrates how the MDEs varies with changes in cluster size. Our findings suggest that, across outcome indicators, the optimal cluster size is around 10 units per cluster. Beyond this point, further increasing the cluster size results in only marginal reduction in MDE, making it less cost-effective relative to the required budget.

**Figure 1**: Changes of Minimum Detectable Effect size (MDE) according to cluster size (number of clusters per arm = 18)

Based on this calculation, our developed our randomization protocol as presented in Figure 3:

**Figure 2:** Randomization protocol

Appendix 3: Covid-19 pandemic development among the Thai and H’Mong communities in 2021

**Figure 4:** Number of COVID-19 cases in Mai Son and Sa Pa in 2021. Data from Mai Son were recorded by the local Women’s Unions at the commune level, and data from 13/17 villages in Sa Pa were recorded by the project implementation team from the Alliance of Bioversity and CIAT.

**Figure 5:** Number of close contacts of Covid-19 cases in Mai Son and Sa Pa in 2021. Data from Mai Son were recorded by the local Women’s Unions at the commune level, and data from 13/17 villages in Sa Pa were recorded by the project implementation team from the Alliance of Bioversity and CIAT.

## Appendix 4: Detailed analysis on Diet diversity and crop diversity

**Figure 6**: The percentage of respondents in the total sample, and disaggregated by ethnicity, consumed the food group in the past 24h. Data collected at baseline

**Figure 7:** The average number of Legumes, Other Vitamin-A rich Vegetables (VitA rich) and Dark Green Leafy Vegetables (DGLV) types (for meaning of this term see footnote in the original paper) grown and consumed in the past 7 days, disaggregated by each food/seed source during baseline.


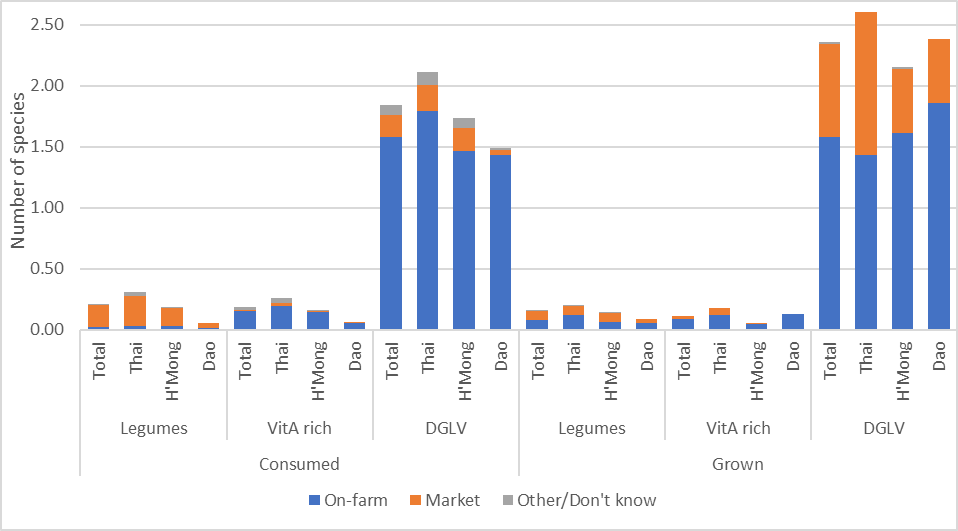

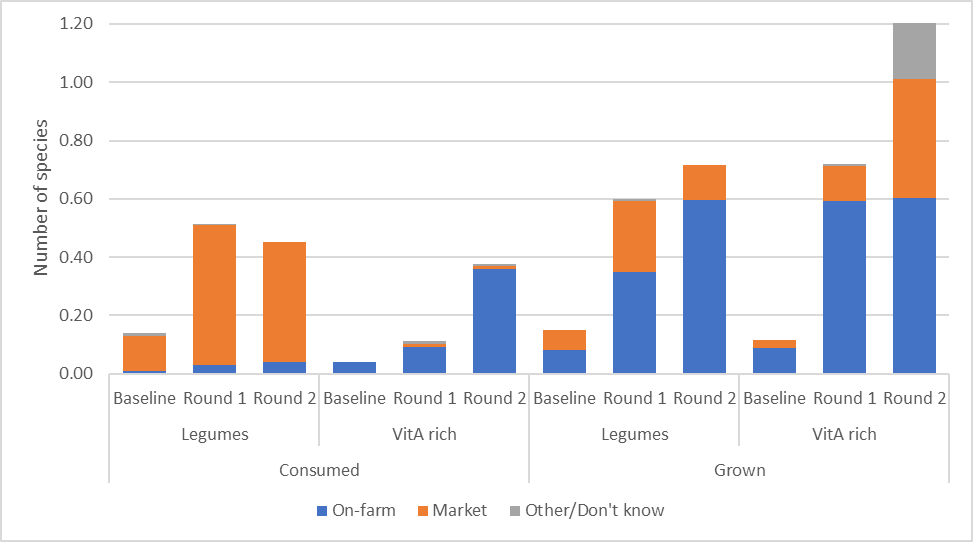


**Figure 8:** The average number of Legumes, Other Vitamin-A rich Fruits and Vegetables (VitA rich) types grown and consumed in the past 7 days, disaggregated by each food/seed source in the baseline and end-line survey rounds. The endline values were computed with the control group only.

## Appendix 5: Balance checks with endline sample and robustness checks for Intent To Treat treatment effects (ITT)

**Table 4:** Mean (SD) at baseline of household and respondent characteristics and outcome variables of the control groups in the total baseline sample, and Thai and H’Mong baseline samples separately; and the coefficients (Robust SE) of the regressions between each dependent variable and treatment assignments of each sample.

| **Dependent variables** | **Total sample** | | | **Thai sample** | | | **H’Mong sample** | | |
| --- | --- | --- | --- | --- | --- | --- | --- | --- | --- |
|  | Control (N=374) | Training (N=596) | Seed provision on top of the training  (N=295) | Control (N=90) | Training (N=127) | Seed provision on top of the training (N=64) | Control (N=91) | Training (N=200) | Seed provision on  top of the training  (N=97) |
|  | **Mean (SD)** | **Coefficients (SE)** | **Coefficients (SE)** | **Mean (SD)** | **Coefficients (SE)** | **Coefficients (SE)** | **Mean (SD)** | **Coefficients (SE)** | **Coefficients (SE)** |
| **Household and respondent characteristics** |  |  |  |  |  |  |  |  |  |
| Household head is female | 0.07 (0.24) | 0.03 (0.02) | -0.01 (0.02) | 0.08 (0.26) | 0.03 (0.04) | 0.02 (0.05) | 0.06 (0.22) | 0.06* (0.03) | -0.03** (0.02) |
| Respondent is female | 0.86 (0.35) | 0.01 (0.04) | 0.04 (0.03) | 0.87 (0.34) | 0.09 (0.07) | 0.02 (0.05) | 0.84 (0.37) | 0.01 (0.06) | 0.02 (0.04) |
| Household head completed primary school or higher | 0.3 (0.45) | -0.06 (0.06) | -0.02 (0.04) | 0.43 (0.48) | 0.01 (0.08) | -0.10 (0.07) | 0.24 (0.42) | -0.13** (0.06) | 0.03 (0.06) |
| Respondent completed primary school or higher | 0.25 (0.43) | -0.05 (0.05) | 0.06 (0.04) | 0.38 (0.49) | -0.09 (0.06) | 0.12* (0.06) | 0.15 (0.36) | 0.03 (0.04) | 0.01 (0.05) |
| Age of Household head | 45.6 (11.7) | 0.1 (1.7) | -0.8 (1.3) | 46.9 (11.0) | 0.0 (2.3) | -2.70* (1.3) | 43.7 (11.5) | 0.7 (2.3) | 0.7 (2.2) |
| Age of respondents | 39.7 (11.6) | -0.2 (1.5) | -0.7 (1.3) | 41.1 (11.3) | 0.8 (2.3) | -3.40* (1.8) | 38.7 (11.8) | -1.5 (2.0) | 0.7 (1.9) |
| Wealth index | 0.06 (1.85) | -0.15 (0.54) | -0.10 (0.20) | 1.61 (1.64) | 0.32 (0.35) | -0.26 (0.32) | -0.84  (1.23) | -0.43 (0.54) | -0.05 (0.15) |
| Number of household members | 5.53 (1.77) | 0.30* (0.16) | -0.03 (0.13) | 5.3 (1.64) | -0.03 (0.23) | -0.07 (0.25) | 5.76 (1.89) | 0.42** (0.16) | -0.05 (0.15) |
| Walking distance to nearest district market (hours) | 2.86 (1.52) | -0.67* (0.38) | -0.08 (0.16) | 3.18 (1.16) | -0.62* (0.31) | 0.11 (0.28) | 2.27 (1.52) | -0.59 (0.75) | -0.23 (0.19) |
| Walking distance to nearest fresh food market (hours) | 0.36 (0.43) | -0.02 (0.11) | 0.00 (0.03) | 0.22 (0.17) | -0.03 (0.04) | -0.06* (0.03) | 0.27 (0.24) | 0.03 (0.07) | 0.03 (0.06) |
| Total farm size (ha) | 1.84 (1.49) | 0.00 (0.32) | -0.02 (0.15) | 2.41 (1.57) | 0.33 (0.46) | -0.27 (0.25) | 1.43 (1.33) | -0.09 (0.39) | -0.09 (0.13) |
| **Outcome indicators** |  |  |  |  |  |  |  |  |  |
| Nutrition knowledge score | 1.46 (2.96) | -0.29 (0.55) | 0.2 (0.33) | 2.93 (3.8) | -0.04 (1.00) | 0.37 (0.81) | 0.48 (1.81) | -0.25 (0.25) | 0.08 (0.2) |
| ***Diet diversity*** |  |  |  |  |  |  |  |  |  |
| Number of key food groups consumed in the past 24h | 1.07 (0.51) | 0.04 (0.06) | 0.03 (0.07) | 1.09 (0.56) | -0.03 (0.11) | 0.15 (0.12) | 1.07 (0.51) | 0.11 (0.08) | -0.04 (0.09) |
| *The number from on-farm production* | 0.88 (0.45) | 0.11 (0.05) | 0.01 (0.05) | 0.85 (0.46) | 0.14 (0.09) | 0.08 (0.07) | 0.88 (0.47) | 0.08 (0.07) | -0.04 (0.07) |
| *The number from market purchase* | 0.18 (0.42) | -0.04 (0.06) | 0.01 (0.04) | 0.26 (0.5) | -0.16* (0.08) | 0.05 (0.08) | 0.17 (0.4) | 0.04 (0.08) | 0 (0.05) |
| Number of key food types consumed in the past 24h | 1.4 (0.81) | 0.09 (0.09) | 0.03 (0.1) | 1.49 (0.86) | -0.07 (0.14) | 0.22 (0.16) | 1.38 (0.81) | 0.24* (0.13) | -0.08 (0.15) |
| *The number from on-farm production* | 1.13 (0.74) | 0.19*** (0.07) | 0.02 (0.08) | 1.11 (0.75) | 0.20* (0.10) | 0.10 (0.12) | 1.15 (0.78) | 0.24** (0.10) | -0.04 (0.12) |
| *The number from market purchase* | 0.19 (0.45) | -0.06 (0.06) | 0.01 (0.04) | 0.27 (0.55) | -0.19* (0.09) | 0.05 (0.08) | 0.17 (0.41) | 0.04 (0.09) | 0.00 (0.05) |
| Number of key food groups consumed in the past 7day | 1.32 (0.65) | -0.02 (0.11) | 0.01 (0.07) | 1.45 (0.69) | -0.06 (0.11) | 0.01 (0.11) | 1.28 (0.63) | -0.07 (0.19) | 0.03 (0.09) |
| *The number from on-farm production* | 1.04 (0.54) | -0.01 (0.07) | 0.04 (0.05) | 1.06 (0.55) | 0.00 (0.09) | 0.11 (0.09) | 1.04 (0.56) | -0.10 (0.13) | 0.03 (0.07) |
| *The number from market purchase* | 0.3 (0.54) | -0.01 (0.09) | -0.05 (0.06) | 0.41 (0.59) | -0.03 (0.09) | -0.08 (0.14) | 0.28 (0.52) | -0.02 (0.14) | -0.03 (0.05) |
| Number of key food types consumed in the past 7day | 2.24 (1.38) | -0.07 (0.26) | 0.08 (0.15) | 2.64 (1.62) | -0.13 (0.27) | 0.08 (0.21) | 2.11 (1.16) | 0.02 (0.41) | 0.10 (0.22) |
| *The number from on-farm production* | 1.77 (1.27) | -0.01 (0.18) | 0.15 (0.12) | 1.99 (1.49) | 0.00 (0.24) | 0.2 (0.21) | 1.68 (1.12) | 0.06 (0.28) | 0.14 (0.18) |
| *The number from market purchase* | 0.35 (0.67) | -0.03 (0.11) | -0.06 (0.08) | 0.47 (0.73) | -0.03 (0.13) | -0.13 (0.18) | 0.33 (0.67) | -0.05 (0.17) | -0.01 (0.07) |
| ***Food quantity*** |  |  |  |  |  |  |  |  |  |
| Amount of key food groups consumed in the past 24h (g) | 123.6 (125.0) | 43.2*** (12.4) | -16.5 (15.4) | 120.5 (102.7) | 12.2 (14) | 35.1* (17.8) | 127.6  (143.8) | 61.7*** (16.1) | -46.6*** (16.6) |
| *The amount from on-farm production (g)* | 101.6 (119.8) | 47.5*** (13.8) | -18.8 (13.2) | 89.3 (86.7) | 24.5* (12.1) | 18.8** (7.4) | 108.4  (142.6) | 60.8*** (20.0) | -40.3*** (17.9) |
| *The amount from market purchase (g)* | 16.1 (45.8) | 0.1 (4.9) | 1.4 (5.2) | 22.8 (58.2) | -5.8 (6.7) | 9.5 (13) | 14.7 (39.1) | 3.8 (7.5) | -2.9 (3.3) |
| ***Crop diversity*** |  |  |  |  |  |  |  |  |  |
| Number of key food groups grown in the past 3 months | 1.20 (0.50) | 0.00 (0.10) | 0.10 (0.10) | 1.27 (0.59) | 0.08 (0.10) | 0.04 (0.13) | 1.15 (0.50) | -0.02 (0.10) | 0.09 (0.06) |
| *Having any of the seed from Self-saving source* | 0.99 (0.56) | -0.02 (0.07) | 0.10 (0.06) | 0.96 (0.65) | -0.04 (0.11) | 0.13 (0.11) | 0.99 (0.48) | 0.00 (0.10) | 0.07 (0.08) |
| *Having any of the seed from market purchase* | 0.55 (0.63) | 0.02 (0.13) | 0.03 (0.08) | 0.79 (0.64) | 0.11 (0.15) | -0.03 (0.12) | 0.42 (0.60) | 0.03 (0.17) | 0.09 (0.11) |
| Number of key types grown in the past 3 months | 2.61 (1.71) | -0.02 (0.27) | 0.38 (0.17) | 2.97 (1.92) | -0.12 (0.31) | 0.34 (0.36) | 2.34 (1.58) | 0.25 (0.38) | 0.43* (0.21) |
| *Seed from Self-saving source* | 1.74 (1.41) | 0.03 (0.18) | 0.33** (0.16) | 1.67 (1.59) | -0.17 (0.26) | 0.39 (0.33) | 1.7 (1.31) | 0.20 (0.25) | 0.26 (0.19) |
| *Seed from market purchase* | 0.85 (1.16) | -0.01 (0.22) | 0.03 (0.11) | 1.26 (1.3) | 0.13 (0.25) | -0.05 (0.14) | 0.62 (1.00) | 0.06 (0.27) | 0.12 (0.17) |

1. Key food groups include Pulses, Dark Green Leafy vegetables, and Other Vitamin-A rich fruits and vegetables
2. * p-value <0.1; ** p-value <0.05; *** p-value <0.01
3. The sample size in the parentheses are number of observations

**Table 5:** Mean (SD) of household and respondent characteristics and outcome variables of the control groups in the total endline sample, and Thai and H’Mong sample seperately; and the coefficients (Robust SE) of the regressions between each dependent variable and treatment assignments of each sample.

| Dependent variables | Total endline sample | | | Thai endline sample | | | H’Mong endline sample | | | |
| --- | --- | --- | --- | --- | --- | --- | --- | --- | --- | --- |
|  | Control (N=452) | Training (N=733) | Seed provision on top of the training (N=355) | Control (N=187) | Training (N=230) | Seed provision on top of the training (N=118) | Control (N=157) | Training (N=304) | Seed provision on top of the training (N=145) |  |
|  | **Mean (SD)** | **Coefficients**  **(SE)** | **Coefficients**  **(SE)** | **Mean (SD)** | **Coefficients (SE)** | **Coefficients (SE)** | **Mean (SD)** | **Coefficients (SE)** | **Coefficients (SE)** |  |
| Household and respondent characteristics |  |  |  |  |  |  |  |  |  |  |
| Household head is female | 0.04 (0.2) | -0.02 (0.02) | -0.02 (0.02) | 0.05 (0.22) | 0.03 (0.04) | 0.02 (0.04) | 0.02 (0.14) | 0.06** (0.02) | -0.04*** (0.01) |  |
| Respondent is female | 0.87 (0.34) | 0.04 (0.03) | 0.04 (0.03) | 0.83 (0.37) | 0.06 (0.06) | 0.03 (0.06) | 0.87 (0.34) | 0.01 (0.04) | 0.02 (0.03) |  |
| Household head completed primary school or higher | 0.34 (0.46) | 0.00 (0.04) | 0.00 (0.04) | 0.44 (0.47) | 0.00 (0.08) | -0.08 (0.08) | 0.32 (0.46) | -0.15*** (0.05) | 0.03 (0.04) |  |
| Respondent completed primary school or higher | 0.24 (0.43) | 0.04 (0.03) | 0.04 (0.03) | 0.36 (0.48) | -0.06 (0.05) | 0.10* (0.05) | 0.12 (0.33) | 0.01 (0.04) | -0.01 (0.04) |  |
| Age of Household head | 45.8 (11.4) | -0.6 (1.3) | -0.6 (1.3) | 48.3(11.1) | -0.3 (2.3) | -2.9** (1.1) | 43.1 (10.3) | 0.3 (1.9) | 0.9 (1.9) |  |
| Age of respondents | 40.9 (12.0) | 0.1 (1.4) | 0.1 (1.4) | 42.9 (11.8) | -0.3 (2.3) | -3.1** (1.3) | 40.1 (12.2) | -2.2 (1.7) | 2.1 (1.9) |  |
| Wealth index | 0.16 (1.88) | -0.04 (0.2) | -0.04 (0.2) | 1.52 (1.69) | 0.31 (0.36) | -0.25 (0.3) | -0.63 (1.45) | -0.5 (0.48) | -0.02 (0.16) |  |
| Number of household members | 5.38 (1.78) | -0.15 (0.11) | -0.15 (0.11) | 5.32 (1.66) | 0.06 (0.24) | -0.15 (0.24) | 5.53 (1.96) | 0.46*** (0.15) | -0.20* (0.10) |  |
| Walking distance to the nearest district market (hours) | 3.36 (1.66) | -0.03 (0.18) | -0.03 (0.18) | 3.6 (1.18) | -0.74** (0.30) | 0.11 (0.26) | 2.89 (1.93) | -0.74 (0.70) | -0.15 (0.23) |  |
| Walking distance to the nearest fresh food market (hours) | 0.36 (0.40) | 0.01 (0.04) | 0.01 (0.04) | 0.26 (0.16) | -0.04 (0.04) | -0.06* (0.03) | 0.26 (0.23) | 0.06 (0.07) | 0.05 (0.05) |  |
| Total farm size (ha) | 1.80 (1.40) | -0.03 (0.31) | -0.04 (0.15) | 2.28 (1.53) | 0.38 (0.44) | -0.31 (0.25) | 1.44 (1.28) | -0.08 (0.34) | -0.09 (0.14) |  |
| Outcome indicators |  |  |  |  |  |  |  |  |  |  |
| Nutrition knowledge score | 1.8 (3.15) | -0.38 (0.55) | -0.06 (0.29) | 2.76 (3.28) | 0.32 (1.00) | -0.02 (0.80) | 1.2 (3.16) | -0.48 (0.62) | -0.19 (0.2) |  |
| *Diet diversity* |  |  |  |  |  |  |  |  |  |  |
| Number of key food groups consumed in the past 24h | 1.02 (0.45) | 0.04 (0.05) | 0.03 (0.05) | 1.07 (0.52) | -0.04 (0.10) | 0.15 (0.11) | 0.99 (0.41) | 0.10 (0.07) | -0.03 (0.07) |  |
| *The number from on-farm production* | 0.82 (0.42) | 0.10** (0.04) | 0.01 (0.04) | 0.78 (0.42) | 0.11 (0.08) | 0.07 (0.06) | 0.83 (0.45) | 0.09 (0.06) | -0.01 (0.06) |  |
| *The number from market purchase* | 0.19 (0.45) | -0.03 (0.05) | -0.01 (0.03) | 0.31 (0.57) | -0.14 (0.08) | 0.04 (0.08) | 0.13 (0.34) | 0.04 (0.07) | -0.03 (0.04) |  |
| Number of key food species consumed in the past 24h | 1.35 (0.79) | 0.1 (0.09) | 0.01 (0.09) | 1.49 (0.92) | -0.06 (0.13) | 0.21 (0.17) | 1.27 (0.68) | 0.23* (0.11) | -0.09 (0.11) |  |
| *The number from on-farm production* | 1.04 (0.69) | 0.19*** (0.06) | 0.02 (0.07) | 1.02 (0.74) | 0.18 (0.09) | 0.12 (0.13) | 1.04 (0.68) | 0.22** (0.09) | -0.04 (0.09) |  |
| *The number from market purchase* | 0.21 (0.5) | -0.05 (0.06) | 0 (0.03) | 0.34 (0.65) | -0.17 (0.09) | 0.04 (0.08) | 0.14 (0.36) | 0.04 (0.07) | -0.02 (0.03) |  |
| Number of key food groups consumed in the past 7day | 1.32 (0.64) | -0.04 (0.10) | 0.02 (0.06) | 1.47 (0.66) | -0.08 (0.12) | 0.01 (0.12) | 1.3 (0.61) | -0.06 (0.14) | 0.04 (0.07) |  |
| *The number from on-farm production* | 1.04 (0.53) | -0.01 (0.06) | 0.05 (0.05) | 1.06 (0.55) | -0.05 (0.08) | 0.12 (0.09) | 1.09 (0.54) | -0.07 (0.10) | 0.05 (0.06) |  |
| *The number from market purchase* | 0.33 (0.51) | -0.03 (0.07) | -0.05 (0.05) | 0.44 (0.56) | -0.03 (0.09) | -0.08 (0.13) | 0.30 (0.50) | -0.04 (0.10) | -0.04 (0.05) |  |
| Number of key food species consumed in the past 7day | 2.29 (1.56) | -0.13 (0.25) | 0.08 (0.13) | 2.75 (1.9) | -0.22 (0.31) | 0.08 (0.20) | 2.06 (1.14) | 0.02 (0.34) | 0.07 (0.19) |  |
| *The number from on-farm production* | 1.77 (1.38) | -0.05 (0.18) | 0.14 (0.11) | 2.03 (1.71) | -0.11 (0.26) | 0.21 (0.19) | 1.61 (1.03) | 0.08 (0.24) | 0.12 (0.16) |  |
| *The number from market purchase* | 0.37 (0.64) | -0.04 (0.09) | -0.05 (0.06) | 0.5 (0.72) | -0.03 (0.13) | -0.12 (0.17) | 0.35 (0.62) | -0.05 (0.12) | -0.02 (0.06) |  |
| *Food quantity* |  |  |  |  |  |  |  |  |  |  |
| Amount of key food groups consumed in the past 24h (g) | 104.5 (95.9) | 52.5*** (18.1) | -21.51 (21.59) | 106.3 (103.5) | 10.3 (12.4) | 38.7** (17.6) | 104.4 (97.2) | 73.7** (25.5) | -49.7 (29.4) |  |
| *The amount from on-farm production (g)* | 80.0 (83.4) | 57.7*** (19.2) | -22.01 (20.74) | 72.8 (76.4) | 22.2** (10.1) | 25.2** (9.5) | 82.9 (94.4) | 74.3** (28.0) | -43.5 (30.6) |  |
| *The amount from market purchase (g)* | 16.4 (40.5) | -1.2 (4.43) | 0.18 (4.34) | 23.1 (49.4) | -6.4 (7.3) | 8.6 (12.8) | 14.5 (35.6) | 2.1 (5.9) | -3.9 (2.4) |  |
| *Crop diversity* |  |  |  |  |  |  |  |  |  |  |
| Number of key food groups grown in the past 3 months | 1.18 (0.53) | -0.01 (0.06) | 0.07 (0.05) | 1.24 (0.58) | 0.01 (0.1) | 0.07 (0.11) | 1.13 (0.47) | 0 (0.07) | 0.06 (0.05) |  |
| *Having any of the seed from Self-saving source* | 0.97 (0.56) | -0.01 (0.06) | 0.08 (0.05) | 0.97 (0.65) | -0.07 (0.1) | 0.13 (0.09) | 0.95 (0.44) | 0.03 (0.08) | 0.04 (0.06) |  |
| *Having any of the seed from market purchase* | 0.53 (0.59) | 0.04 (0.12) | 0.00 (0.06) | 0.7 (0.61) | 0.12 (0.15) | -0.05 (0.11) | 0.4 (0.56) | 0.08 (0.16) | 0.03 (0.08) |  |
| Number of key species grown in the past 3 months | 2.48 (1.74) | 0.01 (0.27) | 0.29* (0.15) | 2.98 (2.04) | -0.16 (0.32) | 0.29 (0.33) | 2 (1.32) | 0.35 (0.33) | 0.27 (0.18) |  |
| *Seed from Self-saving source* | 1.59 (1.39) | 0.05 (0.18) | 0.27** (0.12) | 1.72 (1.68) | -0.19 (0.23) | 0.37 (0.28) | 1.4 (1.12) | 0.22 (0.27) | 0.2 (0.14) |  |
| *Seed from market purchase* | 0.86 (1.18) | -0.01 (0.21) | 0.00(0.09) | 1.2 (1.4) | 0.09 (0.29) | -0.07 (0.13) | 0.58 (0.87) | 0.13 (0.25) | 0.04 (0.13) |  |

1. Key food groups include Pulses, Dark Green Leafy vegetables, and Other Vitamin-A rich fruits and vegetables
2. * p-value <0.1; ** p-value <0.05; *** p-value <0.01
3. The sample size in the parentheses are number of observations

**Table 6:** Intent to treat (ITT) treatment effects estimated by model 2: using the endline sample without correcting for baseline outcomes

|  | Model 2 | | | | | | | | |
| --- | --- | --- | --- | --- | --- | --- | --- | --- | --- |
|  | Total sample | | | Thai sample | | | H’Mong sample | | |
|  | Control (Mean (SD)) | Training | Seed provision on top of the training | Control (Mean (SD)) | Training | Seed provision on top of the training | Control (Mean (SD)) | Training | Seed provision on top of the training |
| Nutrition knowledge score | 2.44 (4.35) | **1.15**** (0.50) | 0.14 (0.34) | 4.61 (5.67) | 1.35 (0.82) | 0.49 (0.71) | 0.64 (1.91) | **0.92***** (0.2) | 0.15 (0.19) |
| Diet diversity |  |  |  |  |  |  |  |  |  |
| Number of key food groups consumed in the past 24h | 1.11 (0.66) | 0.10** (0.05) | 0.00 (0.05) | 1.23 (0.7) | 0.14** (0.06) | -0.01 (0.07) | 1.04 (0.61) | 0.04 (0.07) | -0.04 (0.07) |
| *The number from on-farm production* | 0.80 (0.57) | 0.09* (0.05) | 0.00 (0.04) | 0.78 (0.55) | **0.26***** (0.06) | -0.05 (0.06) | 0.8 (0.59) | -0.03 (0.08) | -0.01 (0.05) |
| *The number from market purchase* | 0.31 (0.55) | -0.04 (0.05) | 0.01 (0.04) | 0.48 (0.64) | -0.18** (0.07) | 0.03 (0.07) | 0.19 (0.45) | 0.05 (0.06) | -0.01 (0.05) |
| Number of key food species consumed in the past 24h | 1.45 (1.01) | 0.18* (0.10) | 0.01 (0.07) | 1.76 (1.14) | 0.26* (0.14) | 0.03 (0.05) | 1.29 (0.87) | 0.03 (0.14) | -0.02 (0.12) |
| *The number from on-farm production* | 1.06 (0.9) | 0.18* (0.09) | 0.00 (0.05) | 1.22 (1.05) | 0.41** (0.14) | -0.03 (0.09) | 0.97 (0.78) | -0.04 (0.13) | 0 (0.08) |
| *The number from market purchase* | 0.31 (0.57) | -0.03 (0.05) | 0.02 (0.04) | 0.49 (0.66) | -0.18** (0.08) | 0.03 (0.07) | 0.19 (0.46) | 0.04 (0.06) | 0.01 (0.05) |
| Number of key food groups consumed in the past 7day | 1.71 (0.75) | -0.02 (0.06) | 0.07* (0.04) | 1.99 (0.69) | -0.12 (0.10) | 0.13 (0.08) | 1.53 (0.76) | 0.01 (0.06) | 0.01 (0.05) |
| *The number from on-farm production* | 1.14 (0.58) | 0.05 (0.04) | **0.06**** (0.03) | 1.22 (0.6) | 0.12 (0.08) | 0.07 (0.07) | 1.05 (0.59) | -0.02 (0.05) | 0.04 (0.03) |
| *The number from market purchase* | 0.69 (0.75) | -0.11* (0.06) | 0.04 (0.04) | 0.94 (0.72) | **-0.30**** (0.10) | 0.10* (0.05) | 0.57 (0.77) | 0 (0.07) | -0.02 (0.06) |
| Number of key food species consumed in the past 7day | 2.93 (1.75) | 0.06 (0.17) | 0.13 (0.10) | 4.02 (1.74) | -0.15 (0.28) | 0.36 (0.21) | 2.29 (1.35) | -0.01 (0.18) | -0.01 (0.11) |
| *The number from on-farm production* | 2.04 (1.46) | 0.23 (0.17) | 0.01 (0.09) | 2.81 (1.66) | 0.32 (0.27) | 0.05 (0.21) | 1.5 (1.03) | 0.01 (0.17) | 0 (0.08) |
| *The number from market purchase* | 0.76 (0.89) | **-0.16**** (0.07) | 0.07 (0.05) | 1.03 (0.87) | -0.37** (0.13) | 0.15* (0.07) | 0.64 (0.91) | -0.04 (0.09) | 0.00 (0.07) |
| Food quantity |  |  |  |  |  |  |  |  |  |
| Amount of key food groups consumed in the past 24h (g) | 197.7 (220.0) | -2.0 (16.9) | 12.6 (17.0) | 210.4 (237.0) | -2.3 (31.4) | 10.4 (23.4) | 214.9 (220.6) | -7.1 (18.1) | 16.6 (26.3) |
| *The amount from on-farm production (g)* | 149.9 (192.2) | 7.6 (14.1) | -2.5 (11.6) | 146.8 (188.1) | 28.8 (28.9) | -7.4 (25.0) | 170.2 (211.3) | -15.0 (14.9) | -0.8 (14.5) |
| *The amount from market purchase (g)* | 36.0 (118.5) | -11.5 (7.3) | 14.9 (10.8) | 57.4 (166.6) | -32.3 (9.9) | 13.4 (10.1) | 24.6 (68.97) | 4.7 (8.1) | 20.4 (19.1) |
| Crop diversity |  |  |  |  |  |  |  |  |  |
| Number of key food groups grown in the past 3 months | 2.09 (0.81) | -0.10 (0.07) | **0.11**** (0.05) | 2.23 (0.76) | -0.10 (0.13) | 0.12 (0.12) | 1.96 (0.85) | -0.03 (0.07) | 0.09 (0.06) |
| *Having any of the seed from self-saved source* | 1.8 (0.83) | -0.09 (0.08) | -0.01 (0.05) | 1.79 (0.82) | -0.01 (0.13) | -0.07 (0.1) | 1.73 (0.84) | -0.03 (0.08) | -0.04 (0.07) |
| *Having any of the seed from market purchase* | 0.78 (0.83) | -0.06 (0.09) | -0.02 (0.08) | 1.18 (0.81) | -0.10 (0.16) | -0.09 (0.2) | 0.57 (0.78) | -0.07 (0.11) | 0.05 (0.06) |
| Number of key species grown in the past 3 months | 5.15 (3.34) | -0.29 (0.42) | 0.32 (0.2) | 7.14 (3.91) | -0.56 (0.82) | 0.12 (0.34) | 3.65 (1.88) | -0.31 (0.18) | **0.46**** (0.18) |
| *Seed from self-saved source* | 3.87 (2.46) | -0.26 (0.3) | -0.11 (0.12) | 4.96 (2.89) | -0.23 (0.61) | -0.24 (0.26) | 2.89 (1.7) | -0.25 (0.19) | -0.08 (0.11) |
| *Seed from market purchase* | 1.13 (1.60) | -0.04 (0.17) | -0.13 (0.13) | 1.89 (1.98) | -0.18 (0.26) | -0.43 (0.25) | 0.69 (1.01) | -0.08 (0.17) | 0.07 (0.12) |
| Model specification |  |  |  |  |  |  |  |  |  |
| N |  | 1220 |  |  | 424 |  |  | 651 |  |
| Control for household and respondents characteristics |  | Yes |  |  | Yes |  |  | Yes |  |
| Control for baseline outcomes |  | No |  |  | No |  |  | No |  |
| Control for missing baseline dummy |  | No |  |  | No |  |  | No |  |

1. Coefficients, Robust standard errors and p-values were retrieved from the model $Y_{ij}^{1}=\alpha+\beta_{1}T_{1}+\beta_{2}T_{2}+ \rho_{1}X_{ij}+ \rho_{2}{{MA}_{ij}+ \varepsilon}_{ij}\left( 2 \right)$, using pooled sample of two endlines, where $T_{1}$ is a treatment dummy for receiving training, and $T_{2}$ is a dummy for receiving seeds on top of the training.
2. (*) denotes model p-value * p-value <0.1; ** p-value <0.05; *** p-value <0.01
3. Coefficients in **bold** indicates those with corrected p-value from multiple hypothesis testing less than 0.1. The p-values were retrieved from Romano-Wolf procedure, which controls the familywise error rate (FWER) for three families: diet diversity, food quantity and crop diversity.

**Table 7:** Intent to treat (ITT) treatment effects estimated by model 3: Using the endline sample correcting for imputed baseline outcomes

|  | Model 3 | | | | | | | | |
| --- | --- | --- | --- | --- | --- | --- | --- | --- | --- |
|  | Total sample | | |  | Thai sample | |  | H’Mong sample | |
|  | Control (Mean (SD)) | Training | Seed provision on top of the training | Control (Mean (SD)) | Training | Seed provision on top of the training | Control (Mean (SD)) | Training | Seed provision on top of the training |
| Nutrition knowledge score | 2.44 (4.35) | 1.19*** (0.42) | 0.20 (0.30) | 4.52 (5.46) | 1.14 (0.75) | 0.62 (0.54) | 0.59 (1.93) | 1.08*** (0.21) | 0.13 (0.23) |
| Diet diversity |  |  |  |  |  |  |  |  |  |
| Number of key food groups consumed in the past 24h | 1.11 (0.66) | 0.09* (0.05) | -0.01 (0.05) | 1.23 (0.7) | 0.14** (0.05) | -0.03 (0.07) | 1.04 (0.61) | 0.02 (0.07) | -0.03 (0.07) |
| *The number from on-farm production* | 0.8 (0.57) | 0.09 (0.05) | 0.00 (0.04) | 0.78 (0.55) | **0.26***** (0.06) | -0.05 (0.06) | 0.8 (0.59) | -0.05 (0.08) | -0.01 (0.05) |
| *The number from market purchase* | 0.31 (0.55) | -0.03 (0.05) | 0.01 (0.04) | 0.48 (0.64) | -0.16** (0.07) | 0.02 (0.06) | 0.19 (0.45) | 0.04 (0.06) | 0 (0.05) |
| Number of key food species consumed in the past 24h | 1.45 (1.01) | 0.17* (0.10) | 0.01 (0.07) | 1.76 (1.14) | 0.26* (0.14) | 0.01 (0.05) | 1.29 (0.87) | 0.02 (0.14) | -0.01 (0.12) |
| *The number from on-farm production* | 1.06 (0.9) | 0.17* (0.09) | 0.00 (0.06) | 1.22 (1.05) | 0.39** (0.15) | -0.04 (0.1) | 0.97 (0.78) | -0.06 (0.13) | 0 (0.08) |
| *The number from market purchase* | 0.31 (0.57) | -0.03 (0.05) | 0.02 (0.04) | 0.49 (0.66) | -0.15** (0.07) | 0.02 (0.06) | 0.19 (0.46) | 0.04 (0.06) | 0.01 (0.05) |
| Number of key food groups consumed in the past 7day | 1.71 (0.75) | -0.03 (0.06) | 0.08* (0.04) | 1.99 (0.69) | -0.13 (0.1) | 0.12* (0.07) | 1.53 (0.76) | 0.01 (0.06) | 0.01 (0.06) |
| *The number from on-farm production* | 1.14 (0.58) | 0.05 (0.04) | 0.06** (0.03) | 1.22 (0.6) | 0.12 (0.08) | 0.05 (0.06) | 1.05 (0.59) | -0.01 (0.05) | 0.04 (0.03) |
| *The number from market purchase* | 0.69 (0.75) | -0.11* (0.06) | 0.05 (0.04) | 0.94 (0.72) | **-0.30***** (0.10) | 0.11* (0.05) | 0.57 (0.77) | 0 (0.07) | -0.01 (0.06) |
| Number of key food species consumed in the past 7day | 2.93 (1.75) | 0.07 (0.17) | 0.13 (0.09) | 4.02 (1.74) | -0.15 (0.28) | 0.36 (0.21) | 2.29 (1.35) | 0 (0.14) | 0 (0.08) |
| *The number from on-farm production* | 2.04 (1.46) | 0.24 (0.17) | 0.00 (0.08) | 2.81 (1.66) | 0.32 (0.28) | 0.02 (0.2) | 1.5 (1.03) | 0.01 (0.14) | 0 (0.07) |
| *The number from market purchase* | 0.76 (0.89) | **-0.16**** (0.07) | 0.08 (0.05) | 1.03 (0.87) | **-0.37***** (0.12) | 0.16** (0.07) | 0.64 (0.91) | -0.04 (0.09) | 0 (0.07) |
| Food quantity |  |  |  |  |  |  |  |  |  |
| Amount of key food groups consumed in the past 24h (g) | 197.7 (210.0) | -4.7 (17.2) | 14.1 (17.1) | 210.4 (237.0) | -2.6 (31.4) | 9.6 (23.6) | 214.9 (220.6) | -10.5 (20.5) | 19.5 (27.2) |
| *The amount from on-farm production (g)* | 149.9 (192.2) | 3.8 (14.7) | -0.5 (11.9) | 146.8 (188.1) | 27.2 (29.4) | -10.0 (24.2) | 170.2 (211.3) | -20.5 (16.9) | 3.5 (15.2) |
| *The amount from market purchase (g)* | 36.0 (118.5) | -11.5 (7.3) | 14.9 (10.8) | 57.4 (166.6) | -32.5*** (9.9) | 13.9 (10.5) | 24.6 (67.0) | 4.4 (8.0) | 20.9 (18.9) |
| Crop diversity |  |  |  |  |  |  |  |  |  |
| Number of key food groups grown in the past 3 months | 2.09 (0.81) | -0.10 (0.07) | **0.10*** (0.05) | 2.23 (0.76) | -0.1 (0.12) | 0.11 (0.11) | 1.96 (0.85) | -0.03 (0.07) | 0.08 (0.06) |
| *Having any of the seed from self-saved source* | 1.8 (0.83) | -0.09 (0.08) | -0.02 (0.05) | 1.79 (0.82) | 0.01 (0.13) | -0.09 (0.10) | 1.73 (0.84) | -0.03 (0.07) | -0.04 (0.06) |
| *Having any of the seed from market purchase* | 0.78 (0.83) | -0.07 (0.09) | -0.02 (0.08) | 1.18 (0.81) | -0.16 (0.17) | -0.10 (0.22) | 0.57 (0.78) | -0.07 (0.10) | 0.04 (0.06) |
| Number of key species grown in the past 3 months | 5.15 (3.34) | -0.27 (0.39) | 0.22 (0.18) | 7.14 (3.91) | -0.5 (0.78) | -0.10 (0.23) | 3.65 (1.88) | -0.32* (0.17) | **0.40**** (0.18) |
| *Seed from self-saved source* | 3.87 (2.46) | -0.25 (0.29) | -0.19 (0.12) | 4.96 (2.89) | -0.13 (0.62) | -0.38 (0.18) | 2.89 (1.7) | -0.26 (0.17) | -0.08 (0.1) |
| *Seed from market purchase* | 1.13 (1.6) | -0.05 (0.16) | -0.13 (0.12) | 1.89 (1.98) | -0.26 (0.28) | -0.47 (0.27) | 0.69 (1.01) | -0.07 (0.14) | 0.07 (0.12) |
| Model specification |  |  |  |  |  |  |  |  |  |
| N |  | 1220 |  |  | 424 |  |  | 651 |  |
| Control for household and respondents characteristics |  | Yes |  |  | Yes |  |  | Yes |  |
| Control for baseline outcomes |  | Yes |  |  | Yes |  |  | Yes |  |
| Control for missing baseline dummy |  | Yes |  |  | Yes |  |  | Yes |  |

1. Coefficients, Robust standard errors and p-values were retrieved from the model $Y_{ij}^{1}=\alpha+\beta_{1}T_{1}+\beta_{2}T_{2}+ \rho_{1}X_{ij}+ \rho_{2}{{MA}_{ij}+ \varepsilon}_{ij}\left( 2 \right)$, using pooled sample of two endlines, where $T_{1}$ is a treatment dummy for receiving training, and $T_{2}$ is a dummy for receiving seeds on top of the training.
2. (*) denotes model p-value * p-value <0.1; ** p-value <0.05; *** p-value <0.01
3. Coefficients in **bold** indicates those with corrected p-value from multiple hypothesis testing less than 0.1. The p-values were retrieved from Romano-Wolf procedure, which controls the familywise error rate (FWER) for three families: diet diversity, food quantity and crop diversity.

## Appendix 6: ITT and LATE of various diet and crop diversity indicators

**Figure 9:** 90% Confidence Intervals of ITT and LATE of the trainings on **nutrition knowledge score** of the total sample, and Thai and H’Mong sub-samples. LATEs were estimated using the instrument variables approach, when participation was instrumented on treatment assignment. Effect sizes were estimated using the baseline sample and ANCOVA estimator.


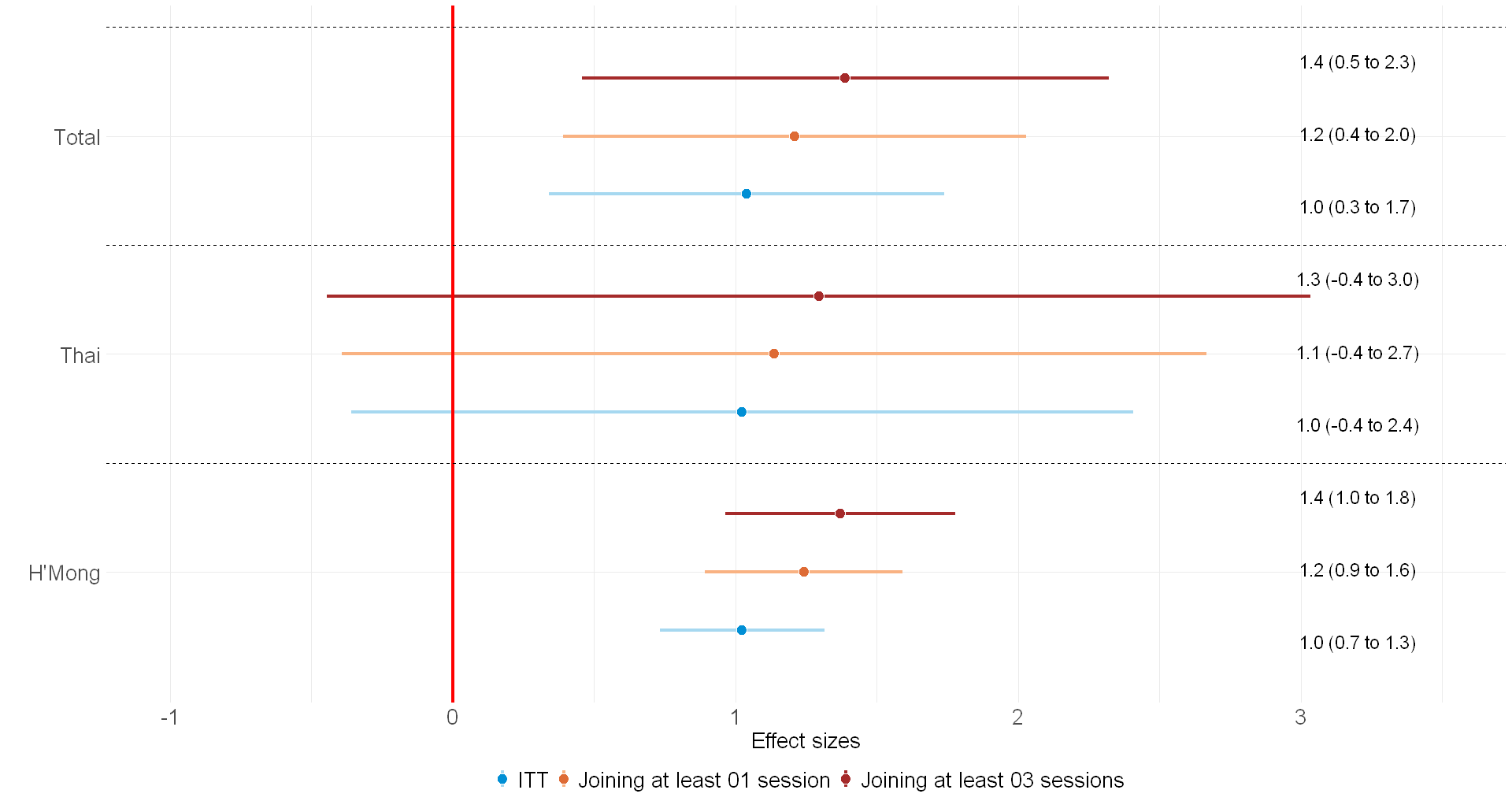

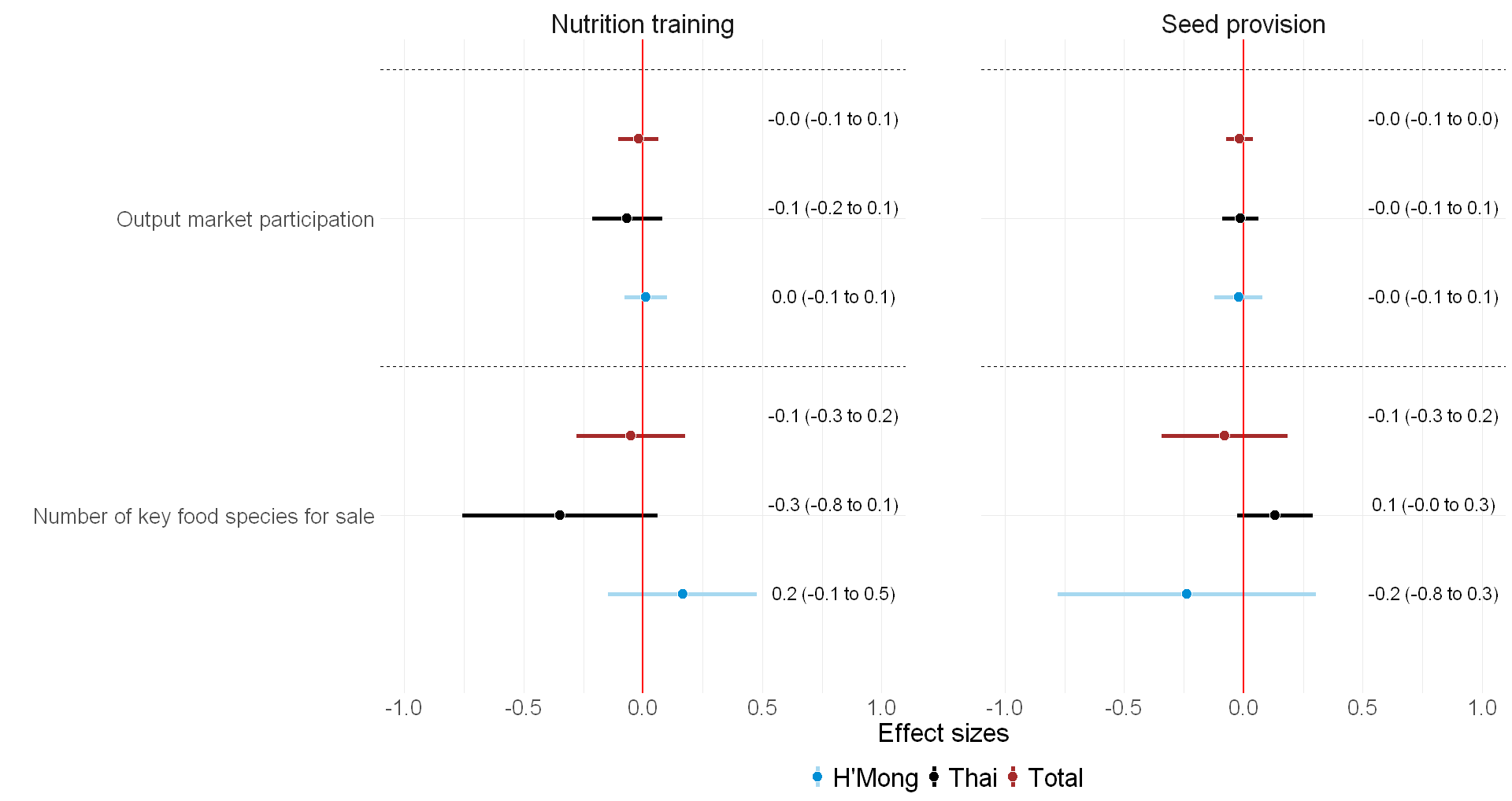


**Figure 10:** 90% Confidence Interval of ITT of Nutrition training and Seed provision on **Output market participation** and **Number of key food species for sale** of the total sample, and Thai and H’Mong sub-samples. Effect sizes were estimated using the baseline sample and ANCOVA estimator. The **Output market participation** indicator is defined as whether a household grows at least one crop whose primary and/or secondary purpose is for sale

Appendix 7: Confidence Intervals and Credential Intervals of ITT

**Figure 12:** 90% Confidence Intervals and 90% Credentials intervals of Intent to treat treatment effects of the **Seed provision on top of the training** estimated using baseline sample and controlling for baseline outcomes. Treatment effects are measured by percentage change from the control mean


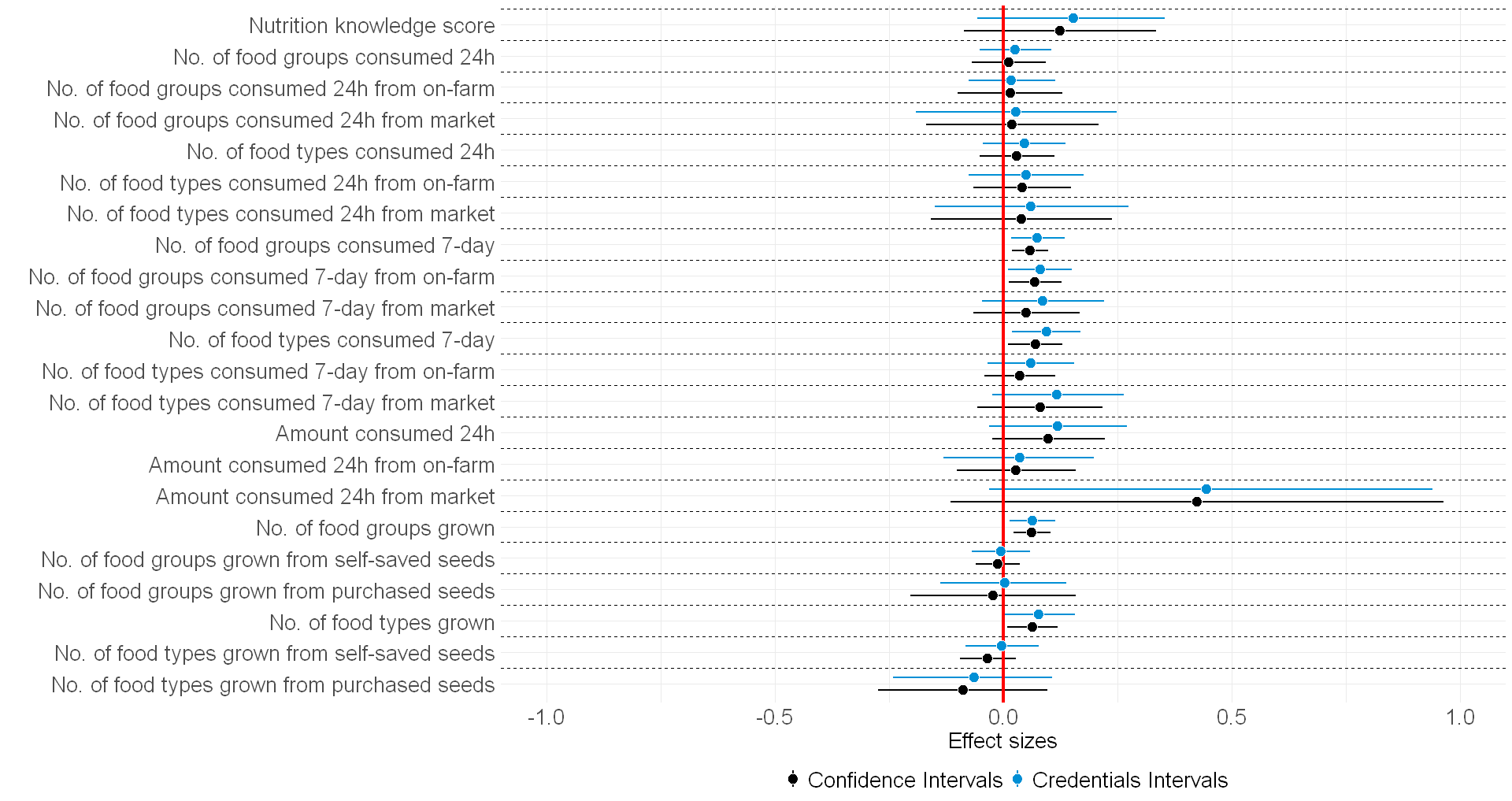


**Figure 11:** 90% Confidence Intervals and 90% Credentials intervals of Intent to treat treatment effects of the **Training** estimated using the baseline sample and controlling for baseline outcomes. Treatment effects are measured by percentage change from the control mean


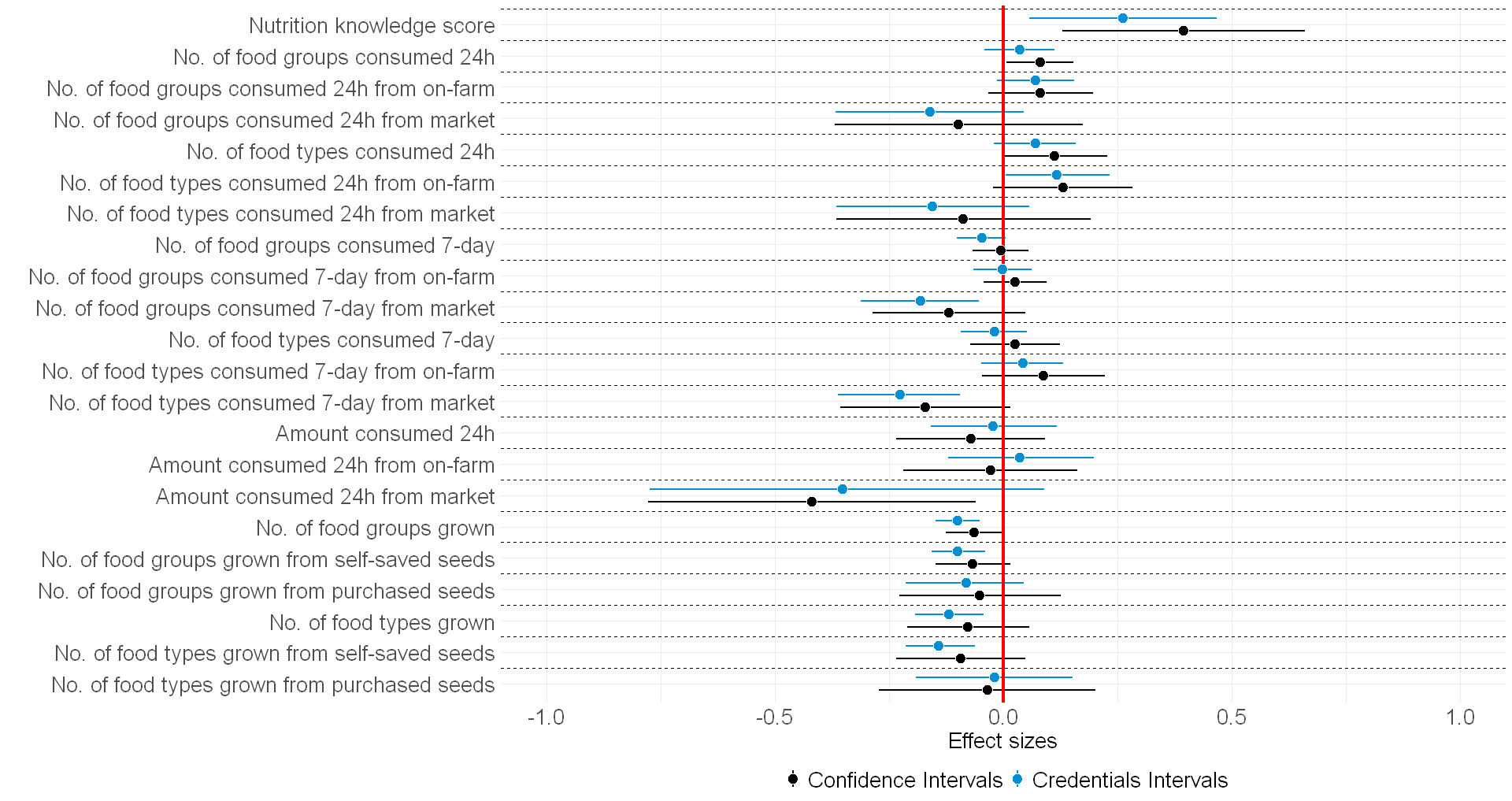

Supplement: Supplementary file 2 — (DOCX 1.04 MB) [file 12571_2025_1580_MOESM2_ESM.docx]
